# Recommended Values of the Fundamental Physical Constants: A Status Report

**DOI:** 10.6028/jres.095.039

**Published:** 1990

**Authors:** Barry N. Taylor, E. Richard Cohen

**Affiliations:** National Institute of Standards and Technology, Gaithersburg, MD 20899; Rockwell International Science Center, Thousand Oaks, CA 91360

**Keywords:** CODATA, conversion factors, electrical units, fundamental physical constants, Josephson effect, least-squares adjustment, quantum Hall effect, recommended values of the constants, Task Group on Fundamental Constants

## Abstract

We summarize the principal advances made in the fundamental physical constants field since the completion of the 1986 CODATA least-squares adjustment of the constants and discuss their implications for both the 1986 set of recommended values and the next least-squares adjustment. In general, the new results lead to values of the constants with uncertainties 5 to 7 times smaller than the uncertainties assigned the 1986 values. However, the changes in the values themselves are less than twice the 1986 assigned one-standard-deviation uncertainties and thus are not highly significant. Although much new data has become available since 1986, three new results dominate the analysis: a value of the Planck constant obtained from a realization of the watt; a value of the fine-structure constant obtained from the magnetic moment anomaly of the electron; and a value of the molar gas constant obtained from the speed of sound in argon. Because of their dominant role in determining the values and uncertainties of many of the constants, it is highly desirable that additional results of comparable uncertainty that corroborate these three data items be obtained before the next adjustment is carried out. Until then, the 1986 CODATA set of recommended values will remain the set of choice.

## 1. Introduction

### 1.1 Background

In late 1986 [1] and also in 1987 [2], CODATA^1^ published a report of the CODATA Task Group on Fundamental Constants prepared by the authors under the auspices and guidance of the Task Group. The report summarizes the 1986 least-squares adjustment of the fundamental physical constants and gives a set of self-consistent values for the basic constants and conversion factors of physics and chemistry derived from that adjustment. Recommended by CODATA for worldwide use throughout all of science and technology and thus widely disseminated [3], the 1986 CODATA set of recommended values replaced its immediate predecessor, that recommended for international use by CODATA in 1973. This set was based on the 1973 least-squares adjustment of the fundamental physical constants which was also carried out by the authors under the auspices and guidance of the Task Group [4,5]. The 1986 adjustment was a major advance over its 1973 counterpart; the uncertainties of the recommended values were reduced by roughly an order of magnitude due to the enormous advances made throughout the precision measurement-fundamental constants field during the 13 years that elapsed between the two adjustments.

Recognizing that the fundamental physical constants field is ever advancing, that is, data affecting our knowledge of the constants are continually appearing, the CODATA Task Group^2^ at its June 1988 meeting asked the authors to prepare a status report on the constants for discussion at its June 1990 meeting. This paper is a direct consequence of that request, which to some extent was motivated by the planned introduction, starting 1 January 1990, of new practical representations of the volt and ohm as defined in the International System of Units or SI. (These new representations will be discussed in sec. 2.1.7.) Another motivating factor was the recognition by the Task Group that 13 years between adjustments is probably too long and that progress in the field should be monitored more closely to help identify when a new set of recommended values should be introduced; the 1973 set had become completely out of date well before the 1986 set was available to replace it.

The 1986 adjustment took into consideration all relevant data available up to 1 January 1986. In the intervening $4\frac{1}{2}$ years, a number of new results have been reported that have important implications for the 1986 CODATA recommended values as well as the timing of the next least-squares adjustment. We summarize these results in this paper and discuss their impact, but do not give new recommended values for any constants. One reason is that because the output values of a least-squares adjustment are correlated, the new results cannot be readily incorporated in the 1986 table of recommended values; to do so properly requires nothing less than a new least-squares adjustment. More important, although the new results can lead to significant reductions in the uncertainties assigned to many of the 1986 recommended values, it is not deemed appropriate to replace the 1986 set so soon after its introduction. There are two reasons for this view. First, it takes considerable time for a new set of recommended values to diffuse throughout all of science and technology; handbooks, text-books, encyclopedias, and other reference works are not revised yearly. Second, the 1986 values adequately serve the needs of the vast majority of users—those few users who require the most up-to-date and accurate values of the constants can consult the primary literature as well as seek advice and guidance from the authors. Based on past experience, it would seem that 6–8 years between adjustments is reasonable; it is not so short an interval that the current set of recommended values has had insufficient time to become widely adopted, or so long that the current set has become totally obsolete. In the final analysis, however, scientific progress should be the deciding factor. If the advances made since the last adjustment would lead to changes in the recommended values several times the one-standard-deviation uncertainties assigned to these values, then a new adjustment may well be immediately called for. If the new results would only lead to reductions in the uncertainties of the recommended values, which as we shall see is the situation at present, then there is considerably less motivation for introducing a new set of values and it is appropriate to wait a longer period. On this basis, we believe that the 1986 set of values should remain the most up-to-date, consistent set available for the next several years and that it will not be necessary to introduce a new set of constants to replace the 1986 set before 1994.

In discussing the new results and their impact, we shall follow to the fullest possible extent the notation, terminology, and order of topics of the 1986 adjustment, reference [2] in particular. To keep this paper to a reasonable length, it is assumed that the reader is familiar with or has reference [2] in hand. After a few brief comments concerning the status of the least-squares evaluation procedure in section 1.2, we review in section 2 the status of the auxiliary constants and stochastic input data. It will be recalled that quantities in the auxiliary constant category are either defined constants such as *c* (speed of light in vacuum = 299 792 458 m/s exactly) and *μ*_0_ (permittivity of vacuum = 4π × 10^−7^ N/A^2^ exactly) with no uncertainty, or constants such as *R*_∞_ (Rydberg constant for infinite mass) with assigned uncertainties sufficiently small in comparison with the uncertainties assigned the stochastic input data with which they are associated in the adjustment that they can be taken as exact. In other words, the auxiliary constants are not subject to adjustment in contrast to the stochastic data. In the 1986 adjustment the uncertainty of each auxiliary constant was no greater than 0.02 parts-per-million or ppm.^3^ In contrast, the uncertainties assigned the 38 items of stochastic input data considered in the 1986 adjustment were in the range 0.065 to 9.7 ppm. The 38 items were of 12 distinct types with the number of items of each type ranging from one to six.

Since this is a status report and not a description of a new least-squares adjustment, our summary of the data in section 2 is not exhaustive and the data are not critically evaluated; we discuss the significant new results only and assume the values and uncertainties as reported are correct. We are therefore addressing the question: If the new results reported since the completion of the 1986 adjustment are taken at face value, what are the implications for the 1986 recommended values? When known, anticipated future results are indicated to provide guidance as to when the next adjustment should be carried out. Where appropriate, the new data are compared with their 1986 counterparts and the 1986 recommended values. The data are further compared and analyzed in section 3, and the implied changes in the 1986 recommended values and their uncertainties as obtained from least-squares analyses that may well preview the next CODATA adjustment are presented in this section as well. Our conclusions are given in section 4.

### 1.2 Data Selection and Evaluation Procedures

Grabe [6] has taken issue with the statistical approaches generally used to treat experimental data, in particular, those employed in the 1986 least-squares adjustment of the constants [7]. He prefers a more conservative approach based on what he terms “abandoning the randomization of systematic errors” [6] that would lead to recommended values of the constants with larger assigned uncertainties. Grabe’s proposed treatment has been extensively rebutted by one of the authors (ERC) in private correspondence and in a brief note [8]. Artbauer [9] has proposed an “interval” approach to the evaluation of measurement uncertainty that, if applied to the least-squares adjustment of the constants, would also likely lead to recommended values with larger uncertainties. At this point, there is little justification for abandoning what has been done in the past; the perceived need by some for recommended values of the constants with “safe” uncertainties was refuted by one of the authors (BNT) 20 years ago [10]. That is not to say that further work to improve the statistical procedures used in a least-squares adjustment should be abandoned; indeed, the authors plan to carry out such work over the next several years with emphasis on refining the statistical techniques used in the 1986 adjustment. But it should be borne in mind that the cornerstone of a successful fundamental constants adjustment is the critical review of each experimental and theoretical result considered for inclusion in the adjustment. Discussions and correspondence with the researchers who have carried out the measurements and calculations are crucial to this process and the evaluator must not accept their *a priori* assigned uncertainties uncritically. By comparison, the particular statistical procedures used in the adjustment play a secondary role.

## 2. Review of the Data

### 2.1 Auxiliary Constants

Because the uncertainties of the auxiliary constants in a least-squares adjustment are generally 10–20 times less than the uncertainties of the stochastic input data, as might be expected, the new results discussed in this section have little impact on the vast majority of the 1986 recommended values. Moreover, it is unlikely that any quantity in the auxiliary constant category in the 1986 adjustment will become a stochastic input datum in the next adjustment.

#### 2.1.1 The Speed of Light and the Definition of the Meter

Principal among the list of recommended radiations given by the International Committee of Weights and Measures (CIPM) [11] for realizing the meter is the He-Ne laser stabilized by saturated absorption on CH_4_ with the adopted frequency *f* = 88 376 181 608 kHz. However, recent measurements [12–15] have shown that this value is too large by about 9 parts in 10^11^, or twice the 4.4 × 10−^11^ uncertainty assigned to it by the CIPM. This implies that the frequencies adopted for the other CIPM recommended radiations, which are in the more important visible portion of the spectrum, are also in error by this amount. Nevertheless, because the smallest uncertainty assigned by the CIPM to these frequencies is 2 parts in 10^10^, the impact is minor. In fact, the only fundamental-constant experiment at present that requires the realization of the meter with an uncertainty of less than 1 part in 10^9^ is the determination of *R*_∞_ (to be discussed in sec. 2.1.4). However, in this case the uncertainty in realizing the meter is the limiting factor.

#### 2.1.2 Proton-Electron Mass Ratio

The 1986 recommended value and that used as an auxiliary constant in the adjustment, *m*_p_/*m*_e_ = 1836.152 701(37) (0.020 ppm), was obtained by van Dyck and colleagues at the University of Washington from Penning-trap ion-cyclotron resonance measurements. It has recently been confirmed to well within the current 0.05-ppm uncertainty of the experiments of Gabrielse and colleagues [16] working at CERN who are using similar techniques but a radically different geometry to measure the antiproton-proton mass ratio [17]. A value of *m*_p_/*m*_e_ with a 0.13-ppm uncertainty that also confirms the 1986 recommended value has been obtained from the H-D isotopic shifts of three transitions as measured in a recent Rydberg constant experiment (see sec. 2.1.4). van Dyck and colleagues are continuing their measurements of *m*_p_*/m*_e_ and believe that the present 0.020-ppm uncertainty can be reduced by an order of magnitude. An improved result from Gabrielse and coworkers may also be expected.

#### 2.1.3 Relative Atomic Masses and Mass Ratios

The 1983 Atomic Mass Table of Wapstra and Audi used in the 1986 adjustment remains the most complete table of values published to date. The 1986 Audi-Wapstra Mid-Stream Mass Evaluation was distributed as a private report [18] and was not fully published [19]. The effect on the fundamental constants of the small differences between the 1986 and 1983 values is negligible. For example, the value of the atomic mass of ^1^H from the 1986 Mid-Stream Mass Evaluation implies the value 1.007 276 468(7) u for the atomic mass of the proton, compared with the 1983 value of 1.007 276 470(12) u. For the atomic mass of the neutron, the corresponding values are 1.008 664 914(8) u and 1.008 664 904(14) u. Advances in cyclotron resonance measurements of single ions in a Penning trap promise to provide improved mass values during the next several years. As an example, van Dyck and colleagues [20] have measured directly the ratio *m*(^12^C^4+^)/*m*_p_ to obtain 1.007 276 468(3) u for the proton atomic mass. A new mass adjustment and atomic mass table to replace that of 1983 is expected to be available in the early 1990s.

The accurate measurement of mass in kilograms is important in a number of fundamental constant experiments, for example, determining the Avogadro constant *N*_A_ by the x-ray crystal density method or determining the Planck constant *h* using a balance that compares electrical and mechanical power. Although the SI unit of mass, the international prototype of the kilogram, has cleaning and stability-related problems [21], these are sufficiently small at present (e.g., ~ 1 part in 10^8^) relative to the uncertainties of such experiments that they can be ignored. However, anticipated improvements in these experiments may confront the kilogram’s limitations and eventually lead to a new definition of the SI unit of mass based on an invariant of nature such as the mass of an elementary particle or atom, or other fundamental constant [22].

#### 2.1.4 Rydberg Constant

**Figure 1:**
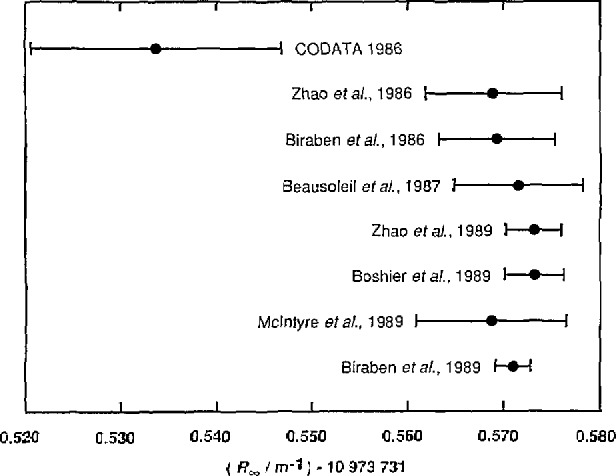
Graphical comparison of the values of the Rydberg constant for infinite mass *R*_∞_ given in table 1.

The 1986 recommended value, *R*_∞_−10 973 731 m^−1^ = 0.534(13) m^−1^, was based to a large extent on the 1981 value (suitably corrected for the new meter definition) 0.539(12) m^−1^ 4 obtained by Amin et al. at Yale from their single photon measurements of the wavelengths of the Balmer-*α* lines in H and D. The experiment was subsequently repeated with a number of improvements, yielding the result 0.569(7) m^−1^ as reported by Zhao et al. in late 1986 [23]. The cause of the difference has yet to be identified. However, a number of other measurements of *R*_∞_ with uncertainties in the parts in 10^10^ range have been reported, and all agree with this higher value (see table 1 and fig. 1).

Biraben et al. [24] at the Ecole Normale Supérieure using Doppler-free, two-photon spectroscopy of H and D Rydberg levels (2*S* − *nD*, *n* = 8,10) obtained 0.569(6) m^−1^. Zhao et al. [25] also measured the 2*S* − 4*P* Balmer-*β* transition in H and D in a modification of their earlier Yale experiment and obtained 0.573(3) m^−1^. Boshier et al. [26] at the University of Oxford measured the 1*S* − 2*S* transition in H and D using Doppler-free, two-photon spectroscopy to find 0.573(3) m^−1^. In a similar experiment, McIntyre et al. [27] at Stanford University obtained 0.569(8) m^−1^. (An earlier version of this experiment at Stanford by Beausoleil et al. [28] in which the uncertainty was assigned more optimistically gave 0.571(7) m^−1^.)

The most recent result and that with the smallest quoted uncertainty,

$$R_{\infty }=10 973 731.5709\left(18\right)m^{-1}\left(1.7\times 10^{-10}\right)$$ (1)

was obtained by Biraben et al. [29,30] from an improved version of their earlier experiment using Rydberg levels. It is this work that has yielded the value of *m*_p_*/m_e_* with an uncertainty of 0.13 ppm mentioned in section 2.1.2. The 1.6 × 10^−10^ uncertainty they assigned to the realization of the meter at visible frequencies based on the 633-nm ^127^I_2_ stabilized laser [11] is the main source of the 1.7 × 10^−10^ relative uncertainty in their value of *R*_∞_; if it could be neglected, the relative uncertainty of their result would be 4 times smaller or 4.3 × 10^−11^.

These and any other *R*_∞_ results that become available will be critically reviewed as part of the next CODATA adjustment. Although it is likely to lead to some changes in values and assigned uncertainties, these should be at the 1–2 parts in 10^10^ level at most and thus not large enough to alter the excellent agreement apparent in table 1 and figure 1. The changes are expected to arise mainly from a uniform treatment of the frequencies assigned to the reference lasers and the theory of the hydrogen atom energy levels.

The recent Biraben et al. value of *R*_∞_ [eq (1)] exceeds the 1986 recommended value by 0.0034 ppm or 2.8 times the 0.0012 ppm uncertainty assigned the 1986 value. Although clearly a significant change, it is sufficiently small relative to the uncertainties of the stochastic input data with which *R*_∞_ was associated in the 1986 adjustment, and the uncertainties of those recommended values derived with the aid of *R*_∞_, that its effect on the 1986 set of values is inconsequential.

#### 2.1.5 *g* Factor of the Free Electron and Muon

The University of Washington group has improved its Penning-trap measurements of the magnetic moment anomalies of the electron and positron: *a*_e_=(µ_e_/µ_B_) − 1 = (*g*_e_/2) − 1, where µ_e_ is the electron magnetic moment and µ_B_ is the Bohr magneton. The new results are [31]

$$a_{e}\left(e^{-}\right)=1 159 652 188.4\left(4.3\right)\times 10^{-12}\left(0.0037\mathrm{ppm}\right)$$ (2a)

$$a_{e}\left(e^{+}\right)=1 159 652 187.9\left(4.3\right)\times 10^{-12}\left(0.0037\mathrm{ppm}\right),$$ (2b)

and *g*_e_(e^−^)/*g*_e_(e^+^) = 1 + (0.5±2.1)×10^−12^. The uncertainties of the two anomalies are dominated by the common 4 × 10^−12^ (0.0034 ppm) uncertainty assigned to each to take into account the possible effect of microwave cavity resonances on the measured cyclotron resonance frequencies. The agreement of the two *g*-factors is the most accurate demonstration to date of charged particle-antiparticle symmetry.

The value of *a*_e_ used in the 1986 adjustment, 1 159 652 193(10) × 10−^12^ (0.0086 ppm), was an earlier result of the University of Washington group but with their originally assigned 4 × 10^−12^ uncertainty (0.0034 ppm) expanded by a factor of 2.5 to allow for the cavity resonance problem. A new method developed to observe these resonances now provides a sound basis for the 4 × 10^−12^ uncertainty included in eq (2) for this effect. The current and earlier results are clearly in excellent agreement. In particular, the 1986 recommended value of *g*_e_/2 exceeds the value implied by eq (2a) by only 5 parts in 10^12^, which is entirely negligible.

Very recent measurements in a new, low-*Q* Penning trap [32] have apparently uncovered a slow magnetic field oscillation induced by a nearby elevator [33]. The simple mean of 14 runs with a non-statistical distribution falling almost uniformally between limits 0.012 ppm apart yields *a*_e_(e^−^)= 1 159 652 185.5(4.0) × 10^−12^, which is still consistent with eq (2). Work to understand the observed distribution of values is continuing.

The 1986 value of *g*_µ_/2 is unchanged. A new experiment to determine *a*_µ_ with an uncertainty of only 0.35 ppm, some 20 times smaller than the current uncertainty, is being undertaken at Brookhaven National Laboratory by V. W. Hughes and collaborators. This will yield a value of *g*_µ_/2 = 1 *+a*_µ_ with an uncertainty of 4.1 parts in 10^10^ compared with the present 8.4 parts in 10^9^.

#### 2.1.6 Electron and Nuclear Magnetic Moment Ratios

The 1986 values of µ_e_/µ_p_, µ_p_/µ_B_, and $\mu \prime_{p}/\mu_{B}$ with their respective 0.010, 0.010, and 0.011 ppm uncertainties remain unchanged. Although µ_e_/µ_B_ is required in the derivation of µ_p_/µ_B_ and $\mu \prime_{p}/\mu_{B}$, its 5 × 10^−12^ increase is too small to have a meaningful effect.

The 1986 recommended value for the deuteron-proton magnetic moment ratio µ_d_/µ_p_ is based on the simple mean of two results: that of Phillips, Kleppner, and Walther (PKW) obtained from the ratio of the g factors for the deuteron and electron in deuterium; and that of Neronov and Barzakh (NB) obtained from the ratio of NMR frequencies in HD. Because the two values differed by more than could reasonably be expected from their *a priori* assigned uncertainties, their simple mean rather than their weighted mean was taken as the recommended value. Recently, Gorshkov et al. [34] carried out new NMR experiments and discovered a systematic error in the NB value. Their new result, µ_d_/µ_p_ = 0.307 012 2081(4), agrees well with that of PKW but has a 10-times smaller uncertainty. It is 0.015 ppm larger than the 1986 recommended value and has an uncertainty that is 13 times smaller than the 0.017 ppm of the 1986 value. This implies that the 1986 recommended values and uncertainties of quantities derived from µ_d_/µ_p_, particularly µ_d_/µ_B_, µ_d_/µ_N_. and µ_d_/µ_e_, will also need to be changed accordingly.

#### 2.1.7 “As Maintained” Volt and Ohm Standards

In the 1986 adjustment, electric unit-dependent quantities such as the Faraday *F* and gyromagnetic ratio of the proton γ_p_ were expressed in terms of the practical laboratory unit of voltage V_76−BI_ defined by the Josephson effect and the adopted value 483 594.0 GHz/V_76−BI_ for the Josephson frequency-voltage quotient; and in terms of the practical laboratory unit of resistance *Ω*_BI85_ defined as the value of *Ω*_69−BI_ on 1 January 1985, where *Ω*_69−BI_ was the time-dependent unit of resistance maintained at the International Bureau of Weights and Measures (BIPM) by means of a group of standard resistors. These noncoherent units were then related to their coherent SI counterparts through the relations V_76−BI_=*K*_v_ V and *Q*_BI85_*=K*_Ω_ *Ω* with the quantities *K*_v_ and *K*_Ω_ taken as unknowns in the least-squares adjustment.

During the years 1986 to 1988, extraordinary advances were made in measuring the Josephson frequency-voltage quotient and quantized Hall resistance (QHR) in SI units and in calibrating laboratory voltage and resistance standards in terms of these quantum effects. These advances led the CIPM, upon the recommendation of its Consultative Committee on Electricity (CCE), to introduce new representations of the volt and ohm for worldwide use starting 1 January 1990 [35,36]. The new representation of the volt is based on the Josephson effect using the value

$$K_{J-90}=483 597.9\mathrm{GHz}/\text{V exactly}$$ (3)

for the Josephson constant *K*_J_, where *U*_J_(*n*)*=nf/K*_J_. (*U*_J_(*n*), *n* an integer, is the voltage of the *n* th constant-current voltage step induced in a Josephson device by radiation of frequency *f*. *K*_J_ is thus the frequency-voltage quotient of the *n* th step times the step number.) Similarly, the new representation of the ohm is based on the (integral) quantum Hall effect using the value

$$R_{K-90}=25 812.807\Omega \;\text{exactly}$$ (4)

for the von Klitzing constant *R*_K_, where *R*_H_(*i*)*=U*_H_(*i*)*/I = R*_K_*/i.* (*R*_H_(*i*) is the quantized Hall resistance of the *i*th plateau, *i* an integer, and is equal to the quotient of the Hall voltage of the *i*th plateau *U_H_*(*i*) divided by the current *I* through the Hall device. *R*_K_ is thus the Hall voltage-current quotient or resistance of the *i*th plateau times the plateau number.)

Equations (3) and (4) imply that *K*_J_ = 483 597.9 GHz/*V*_90_ exactly and *R*_K_ = 25 812.807 *Ω*_90_ exactly, where

$$V_{90}=\left(K_{J-90}/K_{J}\right)V=K_{V}\;V$$ (5)

$$\Omega_{90}=\left(R_{K}/R_{K-90}\right)\Omega =K_{\Omega }\Omega .$$ (6)

The quantities *V*_90_ and *Ω*_90_ are printed in italic type in recognition of the fact that they are physical quantities. (The corresponding quantities were taken as non-SI units in references [1,2].) They are exactly defined by *K*_J−90_ and *R*_K−90_. In practice, laboratory voltage and resistance standards can be calibrated in terms of *V*_90_ and *Ω*_90_ with relative uncertainties considerably less than 0.01 ppm. This is especially true if a Josephson array voltage standard is used [37]; and if the CCE guidelines for making reliable QHR measurements are followed [38] and a cryogenic current comparator is employed [39]. These calibration uncertainties must be included, however, in the total uncertainty assigned any quantity measured in terms of such standards.

Fortunately, expressing electric unit-dependent quantities in terms of *V*_90_ and *Ω*_90_, or in terms of $A_{90}=V_{90}/\Omega_{90},W_{90}=V_{90}^{2}/\Omega_{90}$, and *C*_90_*=A*_90_ s, is a relatively straightforward procedure since most measurements of such quantities that are presently of interest have been carried out in terms of laboratory standards calibrated in terms of, or traceable to, the Josephson and quantum Hall effects. Because *Ω*_69___BI_ is known from the calculable capacitor ohm realizations of the CSIRO/NML [40] to have been varying over the 25 years prior to 1 January 1990 at a constant linear rate given by [41]

$$d\Omega_{69-\mathrm{BI}}/dt=\left(-0.0614\pm 0.0011\right)\mu \Omega /a,$$ (7)

even those few results obtained well before the discovery of the QHE that need to be considered can be reexpressed in terms of *Ω*_90_. (The value given in eq (7) is well supported by the value dΩ_69−BI_/d*t* = (−0.0579±0.0047) µΩ/a obtained from BIPM QHR measurements [42] but differs somewhat from the value used in the 1986 adjustment because of new data and a reevaluation of the older data [40,41].) The drift rates prior to 1 January 1990 of other national resistance units based on precision standard resistors, such as that of the NIST [43,44], are also adequately known for this purpose.

The Josephson and von Klitzing constants are believed to be related to other fundamental constants through

$$K_{J}=2e/h$$ (8)

$$R_{K}=h/e^{2}=\mu_{0}c\alpha^{-1}/2,$$ (9)

where α^−1^ is the inverse fine-structure constant ≃137. Since μ_0_ and *c* are defined constants, in principle a value of *R*_K_ with a given relative uncertainty yields a value of α^−1^ with the same relative uncertainty, and vice versa. It is also useful to note that *K*_J_*R*_K_ = 2/*e* and $K_{J}^{2}R_{K}=4/h$. Equations (8) and (9) were assumed to be exact in the 1986 adjustment and no substantiated experimental or theoretical evidence to the contrary has appeared in the last $4\frac{1}{2}$ years. In fact, considerable new experimental data has been obtained from comparisons of different Josephson and QHE devices that reinforces the view that these equations are correct (see reference [36] for a listing of the appropriate papers). Unless there is a truly startling and unexpected discovery in the next few years, the next set of recommended values of the constants will no doubt also be based on the assumed exactness of these relations.

On the other hand, from a purely physics standpoint, it is of interest to ask the question: What can the fundamental constants tell us about the accuracy of eqs (8) and (9)? One can, of course, compare values of 2*e/h* obtained from appropriate combinations of other constants with values of *K*_J_ obtained from force balance experiments; and values of *α*^−1^ obtained from quantum electrodynamics (QED) with values of *R*_K_ obtained from calculable capacitor ohm realizations. But the more rigorous way to answer this question is to carry out least-squares adjustments that do not assume the equalities expressed in eqs (8) and (9). In such adjustments, *K*_J_ and/or *R*_K_ are taken as phenomenological constants unrelated to *e* and *h.* The adjusted values obtained for them may then be compared with the adjusted values of 2 *e/h* and *h/e*^2^ resulting from the same adjustment. Such considerations are beyond the scope of this report and will not be discussed further. However, they are the subject of a forthcoming paper [45] and will likely be an integral part of the next CODATA adjustment.

The conventional values *K*_J−90_ and *R*_K−90_ [eqs (3) and (4)] recommended by the CCE and adopted by the CIPM were obtained by two CCE working groups from an analysis of all the relevant data available by 15 June 1988. In the analysis, which is thoroughly documented in reference [36], it was assumed for the purpose of including data from measurements of fundamental constants in the derivation of the conventional values of *K*_J_ and *R*_K_ that eqs (8) and (9) are exact. The goal, of course, was to use the best data available to derive values (within certain constraints [36]) that were as close to the SI values as possible so that the new representations would closely approximate the (SI) volt and ohm.

The working groups and the 15 June 1988 cutoff date were established by the CCE at its 17th meeting held in September 1986. The decision of the CCE to proceed with the introduction of new volt and ohm representations based on the Josephson and quantum Hall effects starting 1 January 1990 stimulated the reporting of new and significant results by the cutoff date. In many cases, the new data supplanted similar data used in the 1986 least-squares adjustment. However, as we anticipated (and hoped), the 1986 adjustment has proved to be more reliable than some of its predecessors. *K*_J−90_ exceeds the 1986 recommended value of 2 *e/h* by only 0.47 ppm or 1.6 times the 0.30-ppm one-standard-deviation uncertainty of the 1986 value; and *R*_K−90_ exceeds the 1986 value of *h/e*^2^ by only 0.052 ppm or 1.2 times its 0.045 ppm uncertainty. This reasonable agreement indicates that the new stochastic input data that have become available since the 1 January 1986 cutoff date of the 1986 adjustment will not lead to major changes in the 1986 recommended values. But the new data will lead to significant reductions in the uncertainties of many of these values, a fact not readily apparent from the 0.4-ppm and 0.2-ppm one-standard-deviation uncertainties conservatively assigned by the CIPM and CCE to the ratios *K*_J−90_*/K*_J_ and *R_K_/R*_K−90_, respectively [35,36]. Indeed, these uncertainties are actually larger than the 0.30-ppm and 0.045-ppm uncertainties of the corresponding 1986 recommended values.

#### 2.1.8 Acceleration Due to Gravity

Knowledge of the local value of the acceleration due to gravity *g* is still not a limiting factor in any experiment that requires it, for example, the determination of *K*_J_ by comparing a mechanical force with an electrostatic force using a volt balance. However, anticipated future advances in measuring $h=4/K_{J}^{2}R_{K}$ by comparing mechanical and electrical power using a moving coil balance (to be discussed in sec. 2.2.2) may well require knowing g at the site of the balance with a relative uncertainty of ~3 × 10^−9^. Although modern absolute gravimeters based on either the direct free-fall or symmetrical rise and fall methods are believed to have this capability [46], the results of the second international comparison of absolute gravimeters carried out at the BIPM in 1985 [47] imply an uncertainty 3–5 times larger. The results of the third international comparison conducted at BIPM in 1989 [48] are apparently more encouraging, however.

### 2.2 Primary Stochastic Input Data

Table 2 gives the principal items of stochastic input data of current interest. (See the Appendix for the main laboratory abbreviations used in table 2 and throughout this paper.) Since our purpose is not to carry out a new adjustment of the constants but only to obtain an overview of the impact of the most significant recent results on the 1986 recommended values, as stated in section 1.1, the data are not critically evaluated and our summary of the available data is not exhaustive. This means that the values and uncertainties of some of the listed items may change in the future, and items of data that are only of marginal or historical interest because of their comparatively large uncertainties or because they are known to be in error have been omitted. Further, no attempt has been made to estimate the effective degrees of freedom for each datum as needed for some of the data analysis algorithms used in the 1986 adjustment since the standard least-squares algorithm is deemed adequate for our purpose. Although the new results now available imply that the 12 distinct types of data considered in the 1986 adjustment may be somewhat different in the next adjustment, we discuss them under the 1986 data-type headings for ease of understanding. The following comments apply to the data of table 2, which takes full advantage of the paper by Taylor and Witt [36] documenting the data analysis that led to the values of *K*_J−90_ and *R*_K−90_ adopted by the CCE and CIPM (see sec. 2.1.7). It should also be recognized that some of these data are first results of on-going experiments and eventually will be superseded by newer results.

#### 2.2.1 Direct Ohm Determinations

Data items 1.1, 1.2, and 1.3 in table 2 are the only three currently available, direct, calculable capacitor-based measurements of *R*_K_ in SI units with uncertainties of less than 0.1 ppm. They were reported in 1988 by Small et al. [49] at the CSIRO/NML; Hartland et al. [50] at the NPL; and Shields et al. [43,44] at the NIST. Since *Ω*_90_ = (*R*_K_*/R*_K−90_) *Ω* = *K*_Ω_ *Ω* = (µ_0_*c*α^−1^/2*R*_K−90_) *Ω*, such measurements determine *Ω*_90_ in units of *Ω*, or equivalently *K*_Ω_, and α^−1^. Because of their significantly smaller uncertainties and close ties to QHR measurements carried out in the same laboratories, these three values supplant the five values of *Ω*_BI85_ considered in the 1986 adjustment, including those obtained at the same three laboratories from earlier versions of the same experiments. Omitted from table 2 are the four other independent, similarly obtained values of *Ω*_90_ listed by Taylor and Witt [36] since these have uncertainties that range from 0.22 to 0.61 ppm and would carry negligible weight in any data analysis compared with data items 1.1–1.3.

The three values are in reasonable agreement. Their weighted mean is ${\bar{K}}_{\Omega }=1.000\;000\;028\left(21\right)\left(0.021\;\mathrm{ppm}\right)$, where the uncertainty has been calculated on the basis of internal consistency [2] (i.e., it has not been multiplied by the Birge ratio *R*_B_=(*χ*^2^/ *v*)^1/2^); *χ*^2^ = 2.70 for *v* = 2 degrees of freedom, *R*_B_ = 1.16, and $P_{\chi^{2}}\left(2.70|2\right)≃0.26$. The value of α^−1^ implied by this mean value is

$$\alpha^{-1}\left({\bar{R}}_{K}\right)=137.036 0005\left(32\right)\left(0.021\mathrm{ppm}\right),$$ (10)

a result that exceeds the 1986 recommended value by 0.080 ppm or 1.8 times the 0.045 ppm uncertainty of the 1986 value. Since the uncertainties of the two values only differ by about a factor of 2, it may be concluded that data items 1.1 to 1.3 will influence the 1986 set of recommended values in a significant but not major way. For future reference, we note that the value of *α*^−1^ from the most precise of these data items, that obtained at the NIST, is

$$\alpha^{-1}{\left(R_{K}\right)}_{\mathrm{NIST}}=137.035 9979\left(32\right)\left(0.024\mathrm{ppm}\right).$$ (11)

This result exceeds the 1986 recommended value by 0.061 ppm or 1.4 times its 0.045 ppm uncertainty. Measurements of *Ω*_90_ are continuing at the NIST and additional results may be expected in the next 1–2 years.

#### 2.2.2 Direct Ampere Determinations (now Watt Determinations)

Six values of A_BI85_=V_76−BI_/Ω_BI85_ were initially considered for inclusion in the 1986 adjustment with uncertainties in the range 4.1 to 9.7 ppm. All were eventually discarded because of their disagreement with the other data and/or negligible weight in the adjustment. No new values of this type have become available (i.e., values of *A*_90_ = *V*_90_/*Ω*_90_) or are any expected in the future. Ampere balance experiments have been replaced by more promising volt balance experiments that determine *V*_90_ (see sec. 2.2.3) and watt balance experiments that determine $W_{90}=A_{90}V_{90}=V_{90}^{2}/\Omega_{90}$. The quantity *W*_90_ is an important new input datum since $h=4/K_{J}^{2}R_{K}$, which implies

$$h=4K_{W}/K_{J-90}^{2}R_{K-90},$$ (12)

where

$$W_{90}=K_{W}W=K_{\Omega }^{-1}K_{V}^{2}W.$$ (13)

Thus a measurement of *W*_90_ is actually a measurement of *h* in SI units, (i.e., J s). In combination with the measured value of *Ω*_90_*=K*_Ω_ *Ω*, a determination of *W*_90_ also gives a value of *K*_v_, and thus of *K*_J_=2*e/h* in Hz/V, through the relations

$$K_{V}={\left(K_{\Omega }K_{W}\right)}^{1/2},$$ (14a)

$$K_{J}=2e/h=K_{J-90}/K_{V}.$$ (14b)

Data item 2.1 in table 2 was obtained by Kibble et al. at the NPL [52] from their pioneering moving-coil apparatus that allows one to realize the watt by comparing mechanical and electrical power. A new version of this experiment with the goal of reducing the uncertainty by a factor of 10 is under construction and first results should be available in 1 to 2 years. Data item 2.2 was obtained at the NIST by Olsen et al. [53] using the same method but an apparatus of considerably different geometry. This value is from their initial version of the experiment that used a room temperature, oil-cooled solenoid to generate the required magnetic field. It has now been replaced with a superconducting solenoid that can generate a much larger field and an eventual reduction in uncertainty by a factor of 50 to 100 is hoped for.

Another approach to measuring *W*_90_ is being vigorously pursued at the NRLM and the ETL [54]. It involves comparing mechanical and electrical energy by levitating a superconducting mass with a superconducting coil. Although no result has yet been reported, these researchers believe a relative uncertainty of ∼1 × 10^−8^ is feasible. A similar experiment in underway at the VNIIM.

The two values of *W*_90_ are clearly in good agreement, differing by only 0.33 ppm, but because the NPL datum, item 2.1, has an uncertainty nearly 10 times smaller than that of the NIST datum, item 2.2, the latter will carry negligible weight by comparison. Indeed, the NPL value of *K*_J_, 483 597.903(35) GHz/V (0.073 ppm) [corresponding to *K*_v_ = 0.999 999 994(73) (0.073 ppm)], obtained from eq (14) using NPL data items 1.2 and 2.1, played the dominant role in determining *K*_J−90_ [36]. (The value of *K*_v_ and 2*e/h* implied by the NIST measurements of *W*_90_ and *Ω*_90_, data items 2.2 and 1.3, are from eq (14) 1.000 000 12(67) (0.67 ppm) and 483 597.84(32) GHz/V (0.67 ppm), respectively.) If ${\bar{K}}_{\Omega }$, the weighted mean of data items 1.1 to 1.3 given in section 2.2.1 is used instead of data item 1.2, the NPL value of *W*_90_ yields

$$K_{V}=0.999 999 965\left(69\right)\left(0.069\mathrm{ppm}\right)$$ (15a)

$$K_{J}=2e/h=483 597.917\left(33\right)\mathrm{GHz}/V\left(0.069\mathrm{ppm}\right).$$ (15b)

This value of 2*e/h* exceeds the 1986 recommended value by 0.51 ppm or 1.7 times the 0.30 ppm uncertainty of the 1986 value. More significant, its 0.069 ppm uncertainty is 4.3 times smaller than the 0.30 ppm uncertainty of the latter. The new type 1 and 2 data together will therefore lead to new recommended values of *e*, *h*, *m*_e_, *N*_A_, *F*, and other quantities dependent upon 2*e/h* that differ from the 1986 recommended values by less than twice the uncertainties of the 1986 values, but the uncertainties of the new recommended values will be reduced by more than a factor of four. Indeed, the NPL value of *K*_w_ gives, from eq (12)*h* =6.626 068 21(90) × 10^−34^ J s (0.14 ppm), which is 1.1 ppm less than the 1986 value or 1.8 times the 0.60 ppm uncertainty of the latter. Further, the uncertainty of this value of *h* is 4.4 times smaller than the 0.60 ppm uncertainty of the 1986 value.

#### 2.2.3 Direct Volt Determinations

Data item 3.1 in table 2 is the final result of the liquid-mercury electrometer experiment at the CSIRO/NML of Clothier et al. [55]; a preliminary value with an uncertainty of 0.60 ppm and in good agreement with it was used as an input datum in the 1986 adjustment. Data item 3.2 is the recently reported result from the PTB volt balance experiment of Funck and Sienknecht [56]. Not listed in table 2 is the LCIE kelvin electrometer result with its 2.4 ppm uncertainty, the other direct volt balance determination initially included as an input datum in the 1986 adjustment but later deleted because of its low weight and marginal agreement with the other 1986 data. The value obtained by Bego et al. at the U. Zagreb using a volt balance, which has an assigned uncertainty of 0.35 ppm and was initially considered by the CCE Working Group on the Josephson effect [36] in their analysis of values of *K*_J_, has also been omitted because of its known disagreement with other values and the subsequent identification by Bego and colleagues of several unsuspected systematic errors (see reference [36], Note Added in Proof). This work is continuing and a reliable result with an uncertainty of ~0.3 ppm may eventually be expected.

It is clear that data items 3.1 and 3.2 agree exceedingly well with each other and with the value of *K*_V_ implied by data item 2.1 (the NPL value of *W*_90_) as given in eq (15a); the maximum spread of the three values is less than 0.07 ppm. However, the watt-ohm value has an uncertainty 2.8 times smaller than that of the weighted mean of data items 3.1 and 3.2 $\left[{\bar{K}}_{V}=1.00\;000\;000\left(192\right)(0.192\;\mathrm{ppm}),{\bar{K}}_{J}=483\;597.900\left(93\right)\;\mathrm{GHz}/V\left(0.19\;\mathrm{ppm}\right)\right]$ and as a consequence, carries nearly 8 times as much weight. Hence, although data items 3.1 and 3.2 confirm eq (15) hey have little additional impact on the 1986 recommended values. (The values of *K*_J_ corresponding to data items 3.1 and 3.2 are 483 597.91(13) GHz/V (0.27 ppm) and 483 597.89(13) GHz/V (0.27 ppm), respectively.) Since *K*_J−90_ and *R*_K−90_ were chosen to be consistent with the SI values of *K*_J_ and *R*_K_, *V*_90_, *Ω*_90_, and thus *W*_90_ should be very nearly equal to V, *Ω*, and W (i.e., *K*_v_, *K*_Ω_, and *K*_w_ should all be equal to 1). This is well borne out by the seven data items included under data types 1, 2, and 3.

#### 2.2.4 Faraday Constant

There have been no new results for the Faraday constant since the 1986 adjustment and to the best of our knowledge, no experiments are underway. Data item 4 is the NBS (now NIST) result included in the 1986 adjustment reexpressed in units of *C*_90_*=A*_90_ s=(*V*_90_*/*Ω_90_) s. This was accomplished using the value (−0.0529 ± 0.0040) µΩ/a for the drift rate of *Ω*_NIST_ based on NIST QHR measurements made over the period 1983–1988 [44], and the fact that *Ω*_NIST_ (1 January 1990) = *Ω*_90_−1.69 µΩ [44]. (The electrochemical measurements in the NIST Faraday experiment were carried out in 1975.) This drift rate agrees well with the value (−0.0536±0.0024) µΩ/a reported by Shields et al. [43] based on NIST calculable capacitor ohm realizations in 1973/1974 and 1988. The consistency of the NIST value of *F* with the other data will be discussed in section 3.

#### 2.2.5 Gyromagnetic Ratio (Low Field)

Six values of $\gamma \prime_{p}{\left(\mathrm{lo}\right)}_{\mathrm{BI}85}$ obtained at the ETL, NPL, NIM, NIST, VNIIM, and ASMW with uncertainties in the range 0.24 to 3.25 ppm were initially considered for inclusion in the 1986 adjustment. All but one, that obtained at the NIST with an uncertainty of 0.24 ppm, were eliminated because of their incompatibility with the other data and/or negligible weight in the adjustment. Since 1986, new values from the NIM, NIST, VNIIM, and ASMW have been reported. The new NIST result of Williams et al. [57] (data item 5.1) was obtained from an entirely new apparatus. It is essentially identical to the earlier NIST value, which has an uncertainty of 0.24 ppm or about twice that of the new value. Because the earlier value is not as closely tied to *Ω*_90_ as is the new value, it is not included in table 2. The NIST measurements are continuing and a reduction in the present 0.11-ppm uncertainty is expected. The new VNIIM result of Tarbeev et al. [58,59] (data item 5.2) was obtained after many significant improvements were incorporated in the earlier version of the experiment. It exceeds the previous VNIIM value, which was initially considered for use in the 1986 adjustment and has an assigned uncertainty of 0.62 ppm, by about 5.5 ppm. This work is also continuing.

The new values of $\gamma \prime_{p}\left(\mathrm{lo}\right)$ from the NIM and the ASMW are not listed in table 2 under data type 5 but under data type 7, $\gamma \prime_{p}$ in SI units. This is because the NIM result is not closely tied to a realization of *Ω*_90_, and the ASMW result is not closely tied to either a realization of *V*_90_ or *Ω*_90_. To express the NIM and ASMW $\gamma \prime_{p}\left(\mathrm{lo}\right)$ values in terms of *A*_90_ would require using the results of problematical volt and ohm transfers to the BIPM. Since both laboratories carried out new measurements of $\gamma \prime_{p}$ at about the same time, it is more appropriate to use these to obtain a single value of $\gamma \prime_{p}$ from each laboratory. The relevant equation is

$$\gamma \prime_{p}={\left[\gamma \prime_{p}{\left(\mathrm{lo}\right)}_{\mathrm{LAB}}\;\gamma \prime_{p}{\left(\mathrm{hi}\right)}_{\mathrm{LAB}}\right]}^{1/2},$$ (16)

where the subscript LAB is used to indicate that the practical unit of current *A*_LAB_ in terms of which $\gamma \prime_{p}\left(\mathrm{lo}\right)$ and $\gamma \prime_{p}\left(\mathrm{hi}\right)$ are measured must be the same for both. This laboratory current unit need not be based on the Josephson and quantum Hall effects; any battery and resistor with arbitrary but fixed values may be used to establish *A*_LAB_. Data items 7.1 and 7.2 will be discussed at the end of section 2.2.6 which deals with measurements of $\gamma \prime_{p}\left(\mathrm{hi}\right)$.

The NBS and VNIIM values of $\gamma \prime_{p}\left(\mathrm{lo}\right)$, data items 5.1 and 5.2, are only marginally in agreement; they differ by (0.76±0.38) ppm or 2.0 times the standard deviation of their difference. These values will be compared with the other data in section 3.

#### 2.2.6 Gyromagnetic Ratio (High Field)

The four values of $\gamma \prime_{p}{\left(\mathrm{hi}\right)}_{B185}$ with uncertainties in the range 1.0 to 5.4 ppm considered for inclusion in the 1986 adjustment were obtained at the KhGIMIP, NPL, NIM, and ASMW. Because of inconsistencies with the other data and/or negligible weight, the KhGIMIP and ASMW values were eventually eliminated. As noted in section 2.2.5, new results for $\gamma \prime_{p}\left(\mathrm{hi}\right)$ have been obtained by both the NIM and the ASMW but will be combined with corresponding values of $\gamma \prime_{p}\left(\mathrm{lo}\right)$ and treated as measurements of $\gamma \prime_{p}$. Since no other new $\gamma \prime_{p}\left(\mathrm{hi}\right)$ results are available, the only datum of type 6 listed in table 2 is the NPL value of Kibble and Hunt used in the 1986 adjustment but reexpressed in terms of *C*_90_=*A*_90_ s. This was done by expressing the NPL value in terms of *Ω*_69−BI_ at the time of the measurement (1974), and using the value for dΩ_69−BI_/d*t* given in eq (7) and the fact that *Ω*_69__−BI_(1 January 1990) = *Ω*_90_−1.90 *µ*Ω [60].

The new results for $\gamma \prime_{p}\left(\mathrm{lo}\right)$ and $\gamma \prime_{p}\left(\mathrm{hi}\right)$ obtained at the NIM in 1988 and reported by Liu et al. [61] are

$$\begin{array}{l} \gamma \prime_{p}\left(\mathrm{lo}\right)=26 751.338\left(20\right)\\ \;\times 10^{4}s^{-1}/T_{\mathrm{NIM}-88}\left(0.74\mathrm{ppm}\right) \end{array}$$ (17a)

$$\begin{array}{l} \gamma \prime_{p}\left(\mathrm{hi}\right)=26 751.743\left(42\right)\\ \;\times 10^{4}C_{\mathrm{NIM}-88}/\mathrm{kg}\left(1.55\mathrm{ppm}\right). \end{array}$$ (17b)

These lead, through eq (16), to data item 7.1 in table 2. Similarly, the individual values leading to data item 7.2 obtained at the ASMW in 1985 and reported by Forkert and Schlesok [62] are

$$\begin{array}{l} \gamma \prime_{p}\left(\mathrm{lo}\right)=26\;751.319\left(22\right)\\ \;\times 10^{4}s^{-1}/T_{\mathrm{ASMW}-85}\left(0.81\mathrm{ppm}\right) \end{array}$$ (18a)

$$\begin{array}{l} \gamma \prime_{p}\left(\mathrm{hi}\right)=26 751.534\left(37\right)\\ \;\times 10^{4}C_{\mathrm{ASMW}-85}/\mathrm{kg}\left(1.37\mathrm{ppm}\right). \end{array}$$ (18b)

The two values of $\gamma \prime_{p}$, data items 7.1 and 7.2, are seen to be in poor agreement; they differ by (4.3±1.2) ppm or 3.6 times the standard deviation of their difference. Since *V*_90_ and *Ω*_90_ are very nearly equal to V and *Ω*, the numerical values of $\gamma \prime_{p}\left(\mathrm{lo}\right)$, $\gamma \prime_{p}\left(\mathrm{hi}\right)$, and $\gamma \prime_{p}$ should be nearly equal. With the exception of data item 7.2, and to some extent item 5.2, this is roughly the case. Consequently, item 7.2 and possibly item 5.2 may be inconsistent with the other data as well. This will be investigated further in section 3.

#### 2.2.7 Silicon Lattice Spacing, 2.2.8 Molar Volume of Silicon

At the time of the 1986 adjustment, two values of the *d*_220_ silicon lattice spacing were available and one value of the mean molar volume of silicon *M*(Si)/*ρ*(Si). Because the two *d*_220_(Si) results, one obtained at the NIST and the other at the PTB, were in gross disagreement, they could not be readily combined with each other and the NIST value of *M*(Si)/*ρ*(Si) to obtain a single value of the Avogadro constant *N*_A_ that could be treated as a single stochastic input datum. Rather, the two different types of data were treated separately: the silicon lattice spacing measurements as two items of type 7 data and the molar volume as a single item of type 8 data. In the end, the NIST *d*_220_(Si) value was eliminated because of its severe disagreement with the other data in the adjustment.

During the last 5 years, Deslattes and colleagues [63] at the NIST have discovered unsuspected systematic errors in the original NIST lattice spacing result and have reported [64] a preliminary value based on new data in good agreement with the value from the PTB. Further, the PTB and the CBNM [65] have recently reported a completely independent determination of *M*(Si)/*ρ*(Si). Consequently, the *d*_220_ and *M*(Si)*/ρ*(Si) values of each laboratory as obtained on their own samples now yield two entirely independent values of *N*_A_: a NIST value and a PTB/CBNM value. The relevant equation is

$$N_{A}=\frac{8M\left(\mathrm{Si}\right)}{\rho \left(\mathrm{Si}\right){\left(d_{220}\left(\mathrm{Si}\right)\sqrt{8}\right)}^{3}},$$ (19)

and the two results are given as data items 8.1 and 8.2 in table 2. They agree well with one another, the PTB/CBNM value exceeding that from the NIST by only (0.43 ± 1.62) ppm. These values will be compared to the other data in section 3. Work at the NIST to refine the value of *d*_220_(Si) is continuing and a final result should be available in the near future. The PTB and the CBNM are vigorously pursuing an improved value of *M*(Si)/*ρ*(Si) (the limiting factor in both *N*_A_ results) and a reduction in the uncertainty of their value of *N*_A_ by a factor of 3 to 4 in the next several years seems feasible.

#### 2.2.9 Quantized Hall Resistance

In the 1986 adjustment, six values of the quantized Hall resistance *R*_H_ (i.e., what is now termed the von Klitzing constant or *R*_K_) expressed in terms of *Ω*_BI85_ constituted the type 9 stochastic input data. However, because of the uncertainties associated with the transfer of reference resistors between standards laboratories, QHR measurements that are not tied to a realization of the ohm based on a calculable capacitor and carried out in the same laboratory are not used in this report. With the 1 January 1990 introduction of the new ohm representation based on the QHE and the conventional value *R*_K−90_ for the von Klitzing constant, determining *R*_K_ in terms of a local laboratory unit of resistance defined in terms of a group of precision resistors serves only to calibrate the resistors in terms of the new ohm representation. Consequently, this type of stochastic data provides little useful information as far as the fundamental constants are concerned and will have a limited role to play in future adjustments. Of course, comparing transportable resistors calibrated by different laboratories in terms of *Ω*_90_ can serve as a useful check on the accuracy of each laboratory’s realization of *Ω*_90_. The 1987 international comparison of national resistance standards shows that such realizations are well in hand [41].

#### 2.2.10 Fine-Structure Constant

Our knowledge of the theoretical expression for the electron magnetic moment anomaly *a*_e_ has advanced markedly in the last 3 years due to the Herculean QED calculations of Kinoshita and coworkers [66–68]. Their most recent result is [69]

$$\begin{array}{l} a_{e}\left(\text{theor}\right)=C_{1}\left(\frac{\alpha }{\pi }\right)+C_{2}{\left(\frac{\alpha }{\pi }\right)}^{2}+C_{3}{\left(\frac{\alpha }{\pi }\right)}^{3}+C_{4}{\left(\frac{\alpha }{\pi }\right)}^{4}\\ \;+\cdots +\delta a_{e} \end{array}$$ (20)

with *C*_1_= 1/2, *C*_2_ = −0.328 478 965 …, *C*_3_= 1.176 11(42), *C*_4_ = −1.434(138), and δ*a*_e_ = 4.46 × 10^−12^. *C*_1_ and *C*_2_ have been evaluated analytically, *C*_3_ partly analytically and partly numerically, and *C*_4_ entirely numerically. The small term *δa*_e_ is a sum of contributions arising from muon and tauon loops, and from hadronic and electroweak effects. The total uncertainty in *a*_e_(theor) is 0.0058 ppm, 0.0046 ppm from *C*_3_ and 0.0035 ppm from *C*_4_. (In 1986 the corresponding uncertainties were 0.065, 0.014, and 0.063 ppm.) Together with the improved University of Washington experimental value of *a*_e_ [0.0037 ppm uncertainty, eq (2a)], eq (20) yields the value given as data item 9.1 in table 2,

$$\alpha^{-1}\left(a_{e}\right)=137.035\;992\;22\left(94\right)\left(0.0069\mathrm{ppm}\right),$$ (21a)

which from eqs (6) and (9) is equivalent to

$$K_{\Omega }=0.999\;999\;9672\left(96\right)\left(0.0069\mathrm{ppm}\right).$$ (21b)

The uncertainty of this new QED value of α^−1^ is nearly 10 times smaller than the 0.065 ppm of its predecessor used in the 1986 adjustment and 3.5 times smaller than the 0.024 ppm uncertainty of the next most precise single value of α^−1^ currently available, that given in eq (11) and obtained from the NIST measurement of *R*_K_ (data item 1.3). The two are in reasonable agreement; eq (11) exceeds eq (21a) by (0.041±0.025) ppm or 1.7 times the standard deviation of their difference. Further, the value of *R*_K_ implied by α^−1^(*a*_e_) through *R*_K_*=µ*_0_ *c*α^−1^/2 is only 0.033 ppm smaller than *R*_K−90._ This is not surprising since the NIST value of *R*_K_ and that implied by α^−1^(*a*_e_) from an earlier [36,68] but only slightly different version of eq (20) played a major role in determining *R*_K−90_ [36]. The 0.0069-ppm uncertainty of α^−1^(*a*_e_) is almost small enough to allow α^−1^ to be taken as an auxiliary constant at the present time, but it should be noted that *α*^−1^(*a*_e_) is (0.061 ± 0.022) ppm smaller than $\alpha^{-1}\left({\bar{R}}_{K}\right)$ [eq (10)] or 2.8 times the standard deviation of their difference. Further comparisons of α^−1^(*a*_e_) with the data of table 2 are given in section 3.

Although the new value of α^−1^(*a*_e_) is only 0.020 ppm larger than the 1986 recommended value or 0.44 times the latter’s 0.045-ppm uncertainty, the 0.0069-ppm uncertainty of the new value is 6.5 times smaller than the 0.045 ppm 1986 uncertainty. The implication is that the new value of α^−1^ will not lead to any significant changes in the 1986 recommended values but will lead to a comparable and thus significant reduction in the uncertainties of a number of quantities dependent upon α^−1^, such as the Bohr and nuclear magnetons *µ*_B_ and *µ*_N_ in units of eV/T, the Bohr radius *a*_0_*= α/*4*π R*_∞_, the quantum of circulation *h/*2*m*_e_, and the Compton wavelengths of the electron, proton, and neutron, λ*_c,x_ = h/m_x_c*, x = e, p, or n. Kinoshita and colleagues plan to continue their QED calculations in order to further reduce the uncertainties of the coefficients *C*_3_ and *C*_4_ of eq (20).

Data item 9.2 is a new value of *α*^−1^ from the PTB with an uncertainty of 0.20 ppm as reported recently by Krüger et al. [70]. It is the first really high-precision result from an experiment, underway for many years, to measure λ*v = h/m*_n_, where λ is the wavelength of neutrons of velocity *v*. λ is defined by back reflection from a silicon single crystal of known lattice spacing and *v* is determined using what is essentially a time-of-flight technique. The inverse fine-structure constant is then obtained from

$$\alpha^{-1}={\left[\left(2R_{\infty }/c\right)\left(m_{n}/m_{p}\right)\left(m_{p}/m_{e}\right)\left(h/m_{n}\right)\right]}^{-1/2}.$$ (22)

Using the value of *R*_∞_ given in eq (1), the 1986 recommended values for the auxiliary constants *m*_n_/*m*_p_ and *m*_p_*/m*_e_, and the value *h/m*_n_ = 3.956 0344(16) × 10^−7^ m^2^/s (0.40 ppm) reported by Krüger et al. [70], eq (22) yields the value of *α*^−1^ given in table 2 as data item 9.2,

$$\alpha^{-1}=137.035 993\left(27\right)\left(0.20\mathrm{ppm}\right),$$ (23a)

or equivalently

$$K_{\Omega }=0.999\;999\;97\left(20\right)\left(0.20\mathrm{ppm}\right).$$ (23b)

This result agrees well with all the values of *α*^−1^ and *K*_Ω_ discussed so far but, of course, it has a comparatively large uncertainty. The PTB researchers are continuing their measurements of *h/m*_n_ and hope for some reduction in uncertainty.

Omitted from table 2 is the value of α^−1^ derived from spectroscopic measurements of the fine structure in atomic helium. Considered for inclusion in the 1986 adjustment, it was later eliminated because it was based on an incomplete theoretical expression. Further, its uncertainty was comparatively large and hence it carried negligible weight.

#### 2.2.11 Muon Magnetic Moment

There have been no new developments in this area in the last $4\frac{1}{2}$ years; the two values of *µ*_µ_/*µ*_p_ given in table 2 as data items 10.1 and 10.2 are the same as those used in the 1986 adjustment. V. W. Hughes of Yale University and collaborators working at Los Alamos are undertaking a new version of their earlier experiment to determine the muonium ground-state hyperfine splitting *v*_Mhfs_ from which data items 10.1 and 11.1 were both obtained. A value of α^−1^ with an uncertainty of a few parts in 10^8^ from *µ*_µ_/*µ*_p_ and the experimental value of and theoretical expression for *v*_Mhfs_ is anticipated. First results may be available in the early 1990s.

#### 2.2.12 Muonium Hyperfine Splitting

As for the muon magnetic moment (sec. 2.2.11), there are no new experimental results for the ground-state hyperfine splitting in muonium; the value given in table 2 as data item 11.1 is that used in the 1986 adjustment. On the other hand, there are new theoretical results from a number of workers in the USSR. Some higher-order QED terms have been calculated [71] and the terms evaluated numerically by Sapirstein et al. have now been obtained analytically [72,73]. However, the dominant uncalculated term, that arising from purely radiative corrections and of order *α*^2^(*Zα*)*E*_F_, has yet to be calculated. Its ± 1 kHz estimated limit of error contributes a 0.13-ppm one-standard-deviation uncertainty to data item 11.1, which may be compared with the 0.036-ppm experimental uncertainty. This term must be calculated if the new results expected for *µ*_µ_/*µ*_p_ and *v*_Mhfs_ (see sec. 2.1.11) are to be fully useful. Fortunately, some progress is now being made in its evaluation [74]. Taking *R*_∞_ as given in eq (1), the weighted mean of data items 10.1 and 10.2 for *µ*_µ_/*µ*_p_, and the 1986 recommended value of *µ*_P_/µ_B_, the updated theoretical expression for *v*_Mhfs_ and the experimental value of *v*_Mhfs_ (data item 11.1) yield for the inverse fine-structure constant

$$\alpha^{-1}\left(\mathrm{Mhfs}\right)=137.035 993\left(22\right)\left(0.16\mathrm{ppm}\right),$$ (24a)

or equivalently

$$K_{\Omega }=0.999\;999\;97\left(16\right)\left(0.16\mathrm{ppm}\right).$$ (24b)

This result is identical to that obtained from *h/m*_n_, eq (23), and thus agrees well with all other values. But again, its uncertainty is comparatively large.

### 2.3 Secondary Stochastic Data

No verified existing theory relates the Newtonian constant of gravitation *G* to other fundamental constants, hence its measured values are treated as independent stochastic quantities regardless of the size of their assigned uncertainties. On the other hand, the molar gas constant *R*, Boltzmann constant *k*, and Stefan-Boltzmann constant *σ* are related by the equations

$$k=R/N_{A}$$ (25)

$$\sigma =2\pi^{5}{\left(R/N_{A}\right)}^{4}/15h^{3}c^{2}.$$ (26)

Thus if directly measured values of *R*, *k*, and *σ* with sufficiently small uncertainties are available, they may be included as stochastic input data in a least-squares adjustment on an equal footing with the data discussed in the preceding sections. Although a new result for *R* with an uncertainty of 1.7 ppm has recently been obtained (see sec. 2.3.1), there are no precision measurements of *k* and the directly measured value of *σ* discussed in the 1986 adjustment with its 134 ppm uncertainty remains the best available. Because the situation regarding *k* and *σ* is unlikely to change in the foreseeable future, recommended values of these quantities will continue to be obtained from eqs (25) and (26) with *N*_A_ and *h* taken from the least-squares adjustment and *R* from its directly measured values.

#### 2.3.1 Molar Gas Constant

A new value of the molar gas constant *R* obtained from measurements of the speed of sound in argon at the NIST was reported in 1988 by Moldover et al. [75]. Using a spherical acoustical resonator the volume of which was obtained by weighing the mercury required to fill it, Moldover and colleagues found

R=8.314471(14)J/(mol K)(1.7ppm). (27)

This result is only 4.7 ppm smaller than the 1986 recommended value or less than 0.6 times the 8.4 ppm uncertainty of the latter. (The 1986 recommended value was obtained at the NPL by measuring the speed of sound in argon also, but by means of an acoustical interferometer.) Further, the 1.7 ppm uncertainty of the NIST result is nearly 5 times smaller than the 8.4 ppm uncertainty of the 1986 value. A comparable and thus significant reduction in the uncertainties of other 1986 recommended values that are dependent upon *R*, such as the Boltzmann and Stefan-Boltzmann constants *k* and *σ* [eqs (25) and (26)], the molar volume of an ideal gas *V*_m_, Loschmidt constant *n*_0_*=N*_A_/*V*_m_, and second radiation constant *c*_2_ *= hc/k*, may be expected.

#### 2.3.2 Stefan-Boltzmann Constant

As pointed out in section 2.3, the Stefan-Boltzmann constant must still be obtained indirectly via eq (26). If the new NIST result for *R*, eq (27), is used to evaluate eq (26) along with the 1986 recommended values of *N*_A_ and *h*, then the resulting value of *σ* is 19 ppm less than the 1986 value or about 0.6 times the 34-ppm uncertainty of the latter. Further, this new value of *σ* has an uncertainty of only 6.9 ppm, which is 4.9 times smaller than the 34 ppm of the 1986 value. Only small changes in these figures occur when the values of *N*_A_ and *h* implied by the data of table 2 are used to evaluate eq (26) in place of the 1986 values (see sec. 3.3). One reason is that the product (*N*_A_ *h*)^3^ depends only on auxiliary constants and α^6^, which changes by less than 0.4 ppm; *N*_A_ changes by 1 ppm or less.

#### 2.3.3 Newtonian Constant of Gravitation

No new results for *G* have been reported; the 1986 recommended value remains unchanged. A potentially important experiment is underway at the PTB [76].

## 3. Data Analysis and Results

Our analysis of the data will be limited since, as stated earlier, our purpose is not to obtain new recommended values of the constants but only to survey the impact of recent results on the 1986 set of values. In section 3.1 we briefly study the compatibility of the data items of table 2 using the known relationships among them, a few such comparisons having already been made in previous sections with the aid of eqs (8), (9), (13), and (14). In section 3.2 the data are briefly investigated using the method of least-squares, possibly foreshadowing the next CODATA adjustment. Finally, in section 3.3 we give the changes in the 1986 recommended values and uncertainties of a representative group of constants as implied by the least-squares adjustments of section 3.2.

### 3.1 Relationships Among Data of Different Types

Some preliminary analyses of the stochastic data of table 2 were given in various subsections of section 2.2 and some inconsistencies were identified. For example, the two values of $\gamma \prime_{p}\left(\mathrm{lo}\right)$ differ by 2.0 times the standard deviation of their difference; the two values of $\gamma \prime_{p}$ differ by 3.6 times the standard deviation of their difference; and *α*^−1^(*a*_e_) differs from the value of α^−1^ implied by the weighted mean of the three measurements of *Ω*_90_ by 2.8 times the standard deviation of their difference.

**Figure 2:**
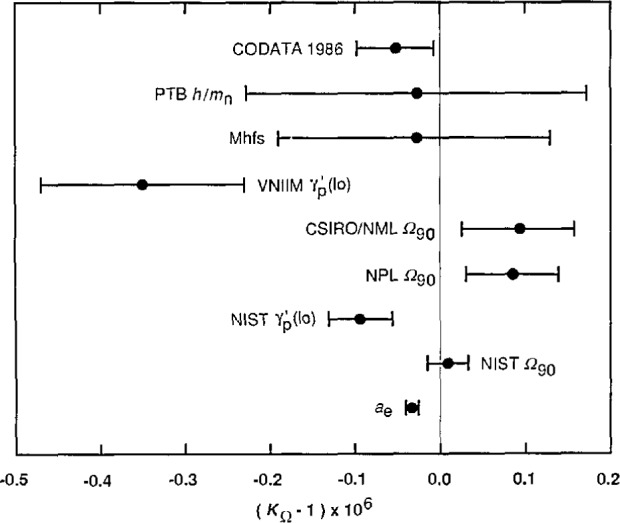
Graphical comparison of the stochastic input data through a comparison of values of *K*_Ω_ = (*R*_K_*/R*_K−90_)*= µ*_0_*cα*^−1^/2*R*_K−90_ given in table 3. With the exception of the 1986 CODATA recommended value, these values are taken directly from or are derived from the stochastic data of table 2.

**Figure 3:**
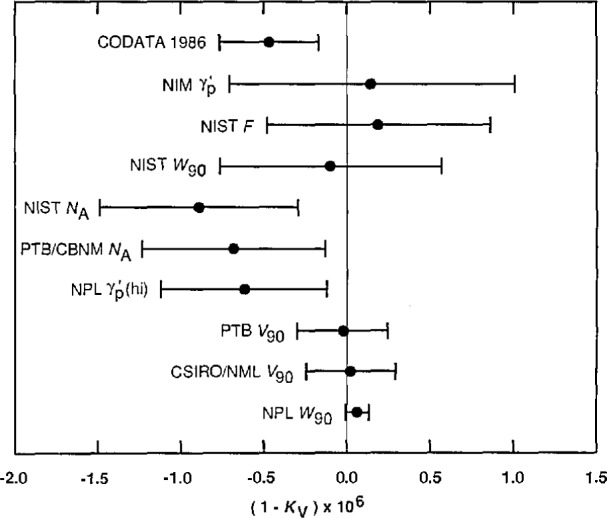
Graphical comparison of the stochastic input data through a comparison of values of *K*_V_=(*K*_J−90_*/K*_J_)*=K*_J−90_*/* (2*e/h*) given in table 4. With the exception of the 1986 CODATA recommended value, these values are taken directly from or are derived from the stochastic data of table 2. (Because its severe disagreement with the other values of *K*_v_ is obvious, value No. 9 of table 4, that derived from the ASMW value of $\gamma \prime_{p}$ (data item 7.2 of table 2), has been omitted from the figure to allow the use of a higher resolution scale.)

An efficient way of further investigating these inconsistencies and of obtaining a clear overview of the compatability of the data of table 2 is to compare the values of *K*_Ω_ and *K*_v_ that the data imply. Table 3, which lists eight values of *K*_Ω_ in order of increasing uncertainty, compares the data of type 1, 5, 9, 10, and 11 in this way; table 4, which lists 10 values of *K*_v_ in order of increasing uncertainty, does the same for the data of type 2, 3, 4, 6, 7, and 8. Because the uncertainties of the data of the latter six types are relatively large compared with the uncertainties of the most precise values of *K*_Ω_, these data cannot provide meaningful values of *K*_Ω_; as far as they are concerned *K*_Ω_, or equivalently α^−1^, is an auxiliary constant. This will be apparent from eqs (29)–(33) to be given shortly in connection with the discussion of table 4. Figures 2 and 3, respectively, graphically compare the values of *K*_Ω_ and *K*_v_ listed in tables 3 and 4. *Note that the values in both the tables and figures are given in the form Δ = (K*_Ω_−1*) ×* 10^6^ *and Δ* = (1−*K*_v_) × 10^6^ *since* Δ is *then the implied ppm change in R*_K−90_ *and K*_J−90_, *respectively.*

The eight values of *K*_Ω_ in table 3 or the values of *α*^−1^ from which they have been derived using the relation *K*_Ω_*=µ*_0_*cα*^−1^/2*R*_K−90_ have already been mentioned in various subsections of section 2.2 except Nos. 3 and 6. These were obtained from the NIST and VNIIM values of $\gamma \prime_{p}\left(\mathrm{lo}\right)$ expressed in units of s^−1^/*T*_90_ (data items 5.1 and 5.2 of table 2) using the relation

$$\alpha^{-1}={\left[\frac{K_{J-90}R_{K-90}\left(\mu \prime_{p}/\mu_{B}\right)}{2\mu_{0}R_{\infty }\gamma \prime_{p}{\left(\mathrm{lo}\right)}_{90}}\right]}^{1/3},$$ (28)

and taking the 1986 recommended value for $\mu \prime_{p}/\mu_{B}$ and eq (1) for *R_∞_.* Because of the comparatively small uncertainties of these two auxiliary constants and the cube root, the uncertainty of the value of α^−1^ and thus of *K*_Ω_ derived from eq (28) is 1/3 that of $\gamma \prime_{p}{\left(\mathrm{lo}\right)}_{90}$. The value of *K*_Ω_ derived from the NIST $\gamma \prime_{p}{\left(\mathrm{lo}\right)}_{90}$ result is thus the third most precise value listed in table 3, but the value from α^−1^(*a*_e_) (No. 1) is still 5.4 times more precise. Indeed, as noted previously, the uncertainty of this value is even 3.5 times smaller than the uncertainty of the next most precise value (No. 2), that obtained from the NIST measurement of *Ω*_90_ (data item 1.3 of table 2). This means that *α*^−1^(*a*_e_) will essentially determine the final value of *K*_Ω_ and thus α^−1^ in any least-squares adjustment in which it is included.

Table 3 and figure 2 show that the other seven values of *K*_Ω_ differ from value No. 1 by less than twice the standard deviation of their difference except No. 4 (NPL *Ω*_90_, data item 1.2) and No. 6 (VNIIM $\gamma \prime_{p}\left(\mathrm{lo}\right)$, data item 5.2), which differ from No. 1 by 2.2 and 2.6 standard deviations, respectively. Thus, while the data are not in gross disagreement, the inconsistencies are clearly larger than one would like.

The values of *K*_v_ listed in table 4 and graphically compared in figure 3, with the exception of the CSIRO/NML and PTB direct measurements of *V*_90_ (Nos. 2 and 3 of table 4, data items 3.1 and 3.2), were obtained using *α*^−l^(*a*_e_) or equivalently, the value of *K*_Ω_ it implies [eq (21b)]. Although there are significant differences among the various values of *K*_Ω_ given in table 3, these are sufficiently small that their effect on the derived values of *K*_v_ is relatively minor. Equation (14a) was used to derive the values of *K*_v_ from the NPL and NIST measurements of *W*_90_ (Nos. 1 and 7, data items 2.1 and 2.2). Written as a relation involving 2*e/h* and *α*^−1^ eq (14a) becomes

$$\frac{2e}{h}={\left[\frac{\mu_{0}c\alpha^{-1}\left(W_{90}/W\right)}{2K_{J-90}^{2}R_{K-90}}\right]}^{-1/2}$$ (29)

The following equation was used to derive the value of *K*_J_ = 2*e/h* and thus *K*_V_ *= K*_J−90_*/K*_J_ from the NIST measurement of the Faraday constant expressed in units of *C*_90_*=A*_90_ s (No. 8, data item 4.1):

$$\;\frac{2e}{h}={\left[\frac{8K_{J-90}R_{K-90}R_{\infty }\alpha^{-1}\left(m_{p}/m_{e}\right)F_{90}}{\mu_{0}c^{2}M_{p}}\right]}^{1/2},$$ (30)

where we have taken the 1986 recommended value for *m*_p_*/m*_e_ and the new University of Washington value for *M*_p_ given in section 2.1.3. The similar equation used to derive the value of 2*e/h* and thus *K*_v_ from the NPL high-field measurement of $\gamma \prime_{p}$ in units of *C*_90_/kg (No. 4, data item 6.1) is

$$\frac{2e}{h}={\left[\frac{8K_{J-90}R_{K-90}R_{\infty }\alpha^{-1}\gamma \prime_{p}{\left(\mathrm{hi}\right)}_{90}}{\mu_{0}c^{2}\left(\mu \prime_{p}/\mu_{B}\right)}\right]}^{1/2}.$$ (31)

The relation employed to derive the values of 2*e/h* from the ASMW and NIM values of $\gamma \prime_{p}$ (Nos. 9 and 10, data items 7.2 and 7.1) is

$$\frac{2e}{h}=\frac{4R_{\infty }\alpha^{-2}\gamma \prime_{p}}{c\left(\mu \prime_{p}/\mu_{B}\right)}.$$ (32)

Finally, the equation used to derive the values of 2*e/h* from the NIST and PTB/CBNM measurements of *N*_A_ (Nos. 5 and 6, data items 8.1 and 8.2) is

$$\frac{2e}{h}={\left[\frac{16R_{\infty }\alpha^{-1}\left(m_{p}/m_{e}\right)N_{A}}{\mu_{0}c^{2}M_{p}}\right]}^{1/2}.$$ (33)

Since the assigned uncertainties of the measured values of the five quantities *W*_90_/W, *F*_90_, $\gamma \prime_{p}{\left(\mathrm{hi}\right)}_{90}$, $\gamma \prime_{p}$, and *N*_A_ lie in the range 0.14 to 1.2 ppm (see table 2), and the assigned uncertainty of α^−1^(*a*_e_) is only 0.0069 ppm, eqs (29)–(33) clearly indicate that as far as these data are concerned, α^−1^(*a*_e_) may be treated as an auxiliary constant.

Table 4 and figure 3 show that the other nine values of *K*_v_ differ from the most precise value, that obtained from the NPL measurement of *W*_90_ (No. 1, data item 2.1), by at most 1.6 times the standard deviation of their difference except No. 9. This value of *K*_v_, obtained from the ASMW measurement of $\gamma \prime_{p}$ (data item 7.2), differs from the NPL *W*_90_ value by 5.2 times the standard deviation of their difference. This is a severe discrepancy and implies that the ASMW datum will likely need to be eliminated. Although the remaining data are consistent, there is a disparity in their uncertainties similar to that of the values of *K*_Ω_ given in table 3. For example, the uncertainty of the most precise value is 4.0 times smaller than the uncertainties of the next two most precise values, the CSIRO/NML and PTB measurements of *V*_90_ (Nos. 2 and 3, data items 3.1 and 3.2). This means, as was noted in section 2.2.3, that the NPL *W*_90_ result will to a large extent determine the value of *K*_v_ and thus *K*_J_ = 2*e/h* in any least-squares adjustment in which it is included.

In summary, we see that the data fall into two groups: those results that mainly determine *K*_Ω_ and those that mainly determine *K*_v_. Each group is dominated by a single value significantly more precise than the other data in the same group, implying that eliminating these other data would have little effect. Each of the dominant values is supported by the other data in its group, although the support is weaker in the *K*_Ω_ case than in the *K*_v_ case. In the next section, the data are further examined by the method of least squares.

### 3.2 Multivariate Analysis of the Data

The *N* = 20 data items of table 2, of 11 distinct types, may be expressed with the aid of auxiliary constants in terms of *M* = 3 adjustable constants or unknowns, namely, *K*_Ω_, *K*_v_, and µ_µ_/µ_p_. The observational equation for each data type, 11 in all, is shown in table 5. (These equations follow from many of the relations already given, for example, eqs (5), (6), (14a), and (28)–(33).) By comparison, in the 1986 adjustment *N* = 38 data items of 12 distinct types were expressed in terms of the five unknowns *α*^−1^, *K*_v_, *K*_Ω_, *d*_220_(Si), and µ_µ_/µ_P_, where *K*_v_=V_76−BI_/V and *K*_Ω_ = *Ω*_BI85_*/*Ω (see sec. 2.1.7). Our decision to include only QHR measurements that are tied to a realization of the ohm based on a calculable capacitor; and the elimination of the discrepancy between the NIST and PTB values of *d*_220_(Si) along with the completion by the PTB and the CBNM of their independent measurement of the silicon molar volume *M*(Si)/*ρ*(Si) (see sec. 2.2.7–2.2.8), have allowed the number of unknowns to be reduced from five to three.

In fact, the number of unknowns or variables could be reduced to two: *K*_Ω_ and *K*_v_. This is because the 0.16 ppm uncertainty [see eq (24a)] of the value of a^−1^ implied by the two direct measuremerits of *µ*_µ_/µ_P_ (data items 10.1 and 10.2) and *v*_Mhfs_ (data item 11.1) is so much larger than the 0.0069 ppm uncertainty of *α*^−1^(*a*_e_) that α^−1^(Mhfs) contributes negligibly to the adjusted value of α^−1^. One could just as well delete data items 10.1, 10.2, and 11.1, determine an adjusted value of α^−1^ from a two-variable *K*_Ω_−*K*_v_ least-squares adjustment, use it and *v*_Mhfs_ to determine a “muonium” value of µ_µ_/µ_p_, and then obtain a weighted mean value for µ*_µ_*/*µ*_p_ from the “muonium” value and two direct values. An even more extreme approach would be to determine *K*_Ω_ from an appropriate weighted mean of the values given in table 3, use this result where needed to derive the values of *K*_v_ given in table 4, and then determine *K*_v_ from an appropriate weighted mean of these values. This is legitimate since, as discussed in section 3.1, α^−1^ may be viewed as an auxiliary constant as far as the data of type 2, 3, 4, 6, 7, and 8 are concerned provided it has a sufficiently small uncertainty. However, because we wish to investigate the effect of deleting various items of stochastic input data, including *α*^−1^(*a*_e_), we treat the data using only three-variable *K*_Ω_−*K*_v_−µ*_µ_/µ*_p_ adjustments.^5^

The standard least-squares algorithm, the only one to be employed in the present study as indicated in the introductory paragraph of section 2.2, yields when applied to the 20 data items of table 2 *X* ^2^ = 54.1 for *v = N*−*M* = 17 degrees of freedom; *R*_B_=1.78 and $P_{\chi^{2}}\left(54.1|17\right)≃1\times 10^{-5}$. This large value of *χ*^2^ is due in large part to data item 7.2, the ASMW value of $\gamma \prime_{p}$, which was previously shown to be discrepant; it is responsible for 49 percent of *χ*^2^ but contributes only 0.6 percent to the determination of the adjusted value of $\gamma \prime_{p}$. When deleted, *χ*^2^ is reduced to 27.6 for *v* = 16, *R*_B_= 1.31, and $P_{\chi^{2}}\left(27.6|16\right)≃0.036$. The dominant contributor to *χ*^2^ is now data item 5.2, the VINIIM value of $\gamma \prime_{p}{\left(\mathrm{lo}\right)}_{90}$; it is responsible for 25 percent of *χ*^2^ but contributes less than 0.3 percent to the determination of $\gamma \prime_{p}{\left(\mathrm{lo}\right)}_{90}$. When it is deleted, *χ*^2^ becomes 20.7 for *v* = 15, *R*_B_ = 1.17, and $P_{\chi^{2}}\left(27.6|15\right)≃0.15$. This adjustment, which will be referred to as Adjustment No. 1, gives the following values for the three adjustable constants or unknowns *K*_Ω_, *K*_v_, and µ*_µ_/µ*_P_:

$$K_{\Omega }-1=\left(-0.0285\pm 0.0064\right)\mathrm{ppm}$$ (34a)

$$1-K_{V}=\left(0.026\pm 0.062\right)\mathrm{ppm}$$ (34b)

$$\mu_{\mu }/\mu_{p}=3.183\;345\;48\left(40\right)\left(0.13\;\mathrm{ppm}\right),$$ (34c)

where the uncertainties have been computed on the basis of internal consistency. That is, they have not been multiplied by *R*_B_, an approach followed throughout this and the next section in order not to allow the relatively minor inconsistencies in the data mask or distort the impact of the new results on the 1986 recommended values and their uncertainties. Further to this aim, we do not follow the principle used in the 1986 adjustment and delete data items that contribute in a marginal way to a particular adjustment (e.g., less than a few percent to the determination of their own value). If we did so, it would require deleting different items for different adjustments, thereby clouding the comparison of their results. For example, in Adjustment No. 4 to be discussed below, only data item 9.2 would be deleted on this basis while in Adjustment No. 1, data items 1.1, 1.2, 2.2, 4.1, 6.1, 7.1, 8.1, 8.2, and 9.2 would be so deleted.

Equations (34a) and (34b) imply that the new representation of the ohm based on the QHE and *R*_K−90_ is smaller than the (SI) ohm by (0.0285±0.0064) *µ*Ω; and that the new representation of the (SI) volt based on the Josephson effect and *K*_J−90_ is smaller than the (SI) volt by (0.026±0.062) *µ*V. These differences, inconsequential as far as present day electrical metrology is concerned, imply that *R*_K−90_ would need to be decreased by 0.029 ppm and *K*_J−90_ would need to be increased by 0.026 ppm to bring the new ohm and volt representations into exact conformity with the presently available data as treated in Adjustment No. 1.

It is of interest to investigate the impact of deleting the two dominant data items, the NPL value of *W*_90_ and α^−1^(*a*_e_) (data items 2.1 and 9.1), first separately and then together. The results are given in table 6, along with those from Adjustment No. 1. Data items 7.2 and 5.2 remain sufficiently discrepant that they must also be deleted from Adjustment Nōs. 2–4. An indication of the dominant role played by the NPL value of *W*_90_ and α^−1^(*a*_e_) is the significantly smaller uncertainties of the values of *K*_Ω_ and *K*_v_ resulting from Adjustment No. 1 compared with the uncertainties of the values resulting from Adjustment No. 4 (2.8 and 2.5 times smaller, respectively); and the significant differences in the values of *K*_Ω_ and *K*_v_ themselves resulting from the two adjustments (0.027 ppm and 0.18 ppm for *K*_Ω_ and *K*_v_, respectively, or 4.3 and 2.9 times the uncertainties of these quantities resulting from Adjustment No. 1).

Table 6 also clearly shows the independence of the two groups of data as discussed in connection with tables 3 and 4: deleting the NPL value of *W*_90_ has essentially no effect upon *K*_Ω_ and thus α^−1^ (compare Adjustment No. 2 with No. 1), and deleting α^−1^(*a*_e_) has only a minor effect on *K*_v_ and thus 2*e/h* (compare Adjustment No. 3 with No. 1); deleting both yields nearly the same values of *K*_Ω_ and *K*_v_ obtained when they are deleted separately (compare Adjustment No. 4 with Nos. 2 and 3).

### 3.3 Changes in the 1986 Recommended Values and Their Uncertainties

Table 7 gives the changes in the 1986 recommended values and uncertainties of an important and representative group of fundamental constants as implied by Adjustment Nos. 1–4 of table 6 discussed in the previous section (the molar gas constant *R*, Boltzmann constant *k*, and Stefan-Boltzmann constant *σ* are discussed in sections 2.3, 2.3.1, and 2.3.2). The values and uncertainties of these constants are calculated in the usual way [2,5] from the adjusted values of the unknowns *K*_Ω_, *K*_v_, and µ_µ_/µ_p_ resulting from the indicated adjustment, their variances and covariances, and auxiliary constants as appropriate. Similar patterns of behavior are observed among these constants because they depend on *K*_Ω_ and *K*_v_ in a similar way. For example, because both *F* and $\gamma \prime_{p}$ are directly proportional to $K_{\Omega }^{-2}-K_{v}^{-1}$, the entries for these quantities in all four columns of each of the four adjustments are nearly identical. Similarly, since the electron Compton wavelength $\lambda_{C}\;\propto \;K_{\Omega }^{-2}$ and α^−1^ ∝ *K*_Ω_, the entry for λ_c_ in column 1 of each adjustment is essentially −2 times the entry in column 1 for α^−1^; the entry in column 2 for λ_c_ is −1 times the entry in column 2 for α^−1^; the entry in column 3 for λ_c_ is 2 times the entry in column 3 for α^−1^; and the entries in column 4 are the same for both λ_c_ and α^−1^. If we had chosen to include in table 7 other constants that can be expressed in terms of the fine-structure constant (and auxiliary constants), such as the Bohr radius, the quantum of circulation, the Compton wavelengths of the proton and neutron, the classical electron radius, and the Thomson cross section [2], their entries would have followed a pattern related to that of α^−1^. In particular, all would show the same 6.9 times reduction in uncertainty characteristic of α^−1^ and λ_c_ in Adjustment No. 1.

The comparatively small change in the value of the muon-electron mass ratio *m*_μ_*/m*_e_ across the four adjustments in table 7 reflects the similar small change in *μ_μ_*/*μ*_p_ in table 6. This is because *m_μ_/m*_e_=(*μ*_e_*/μ*_p_)(*g*_μ_*/g*_e_)/(*μ_μ_/μ*_p_) depends only on *μ_μ_*/μ_p_ and auxiliary constants. The ratio μ_μ_/μ_P_ is determined to some extent (about 18 percent) by the two direct measurements (data items 10.1 and 10.2 of table 2) which were also used in the 1986 adjustment, but to a much greater extent (82 percent) by the “muonium” value of μ_μ_/μ_p_ determined from α^−1^ and *v*_Mhfs_ (see sec. 3.2). Because the current “muonium” value is very nearly equal to its 1986 value and to the weighted mean of the two direct measurements [2], the variation in µ_µ_/µ_p_ and thus in *m*_µ_*/m*_e_ is unusually small.

Many of the other patterns displayed in table 6 are apparent in table 7. For example, this table once again shows that the constants fall into two groups: those strongly dependent upon α^−1^ or equivalently, *K*_Ω_, and those strongly dependent upon *K*_v_. Deleting the NPL value of *W*_90_ (see Adjustment No. 2), which plays the dominant role in determining *K*_v_, has little impact on the values of those constants determined by *K*_Ω_, for example, λ_c_; and deleting *α*^−1^(*a*_e_) (see Adjustment No. 3), which plays the dominant role in determining *K*_Ω_, has little impact on the values of the constants determined to a large extent by *K*_v_, for example *m*_e_.

**Figure 4:**
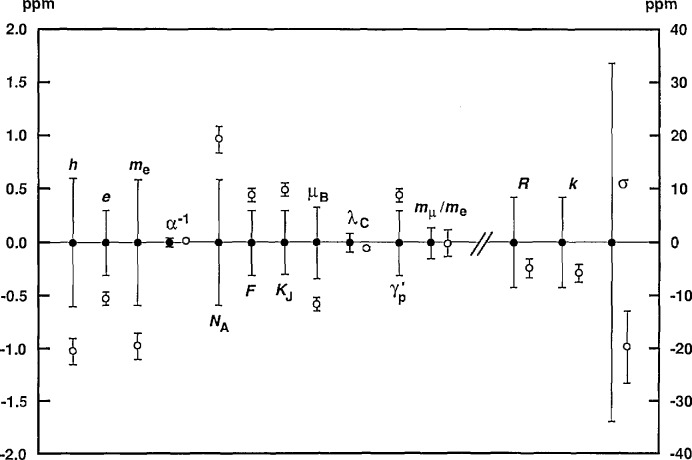
Graphical display of the results of Adjustment No. 1 given in table 7. The closed circles with error bars indicate the 1986 CODATA recommended values and their one-standard-deviation uncertainties; the open circles with error bars indicate the values and their one-standard-deviation uncertainties derived from the data of table 2 through Adjustment No. 1, relative to the 1986 values.

Because table 7 is relatively self explanatory, it does not require a great deal of additional comment. It is clear that the changes in the 1986 recommended values as given by Adjustment No. 1 (depicted in fig. 4), the most important case since it includes the two most precise items of stochastic input data, are significant in comparison with the uncertainties of the 1986 values but not disturbingly so (see column 2). Indeed, in light of recent past adjustments [2,4,77], changes of less than two standard deviations are a welcome sight. In contrast, the reductions in the uncertainties of the 1986 values resulting from Adjustment No. 1 (see column 4) are clearly major and perhaps even extraordinary inasmuch as only $4\frac{1}{2}$ years have elapsed since the 1 January 1986 cutoff date of the 1986 adjustment. The NPL value of *W*_90_, the new University of Washington-Cornell value of *α*^−1^(*a*_e_), and the NIST value of *R*, all obtained in the last 2–3 years, are principally responsible for the large reduction factors. These factors are typically in the range 4.7 to 6.9. Without these three new results, the changes in the 1986 values are far less significant and the reductions in their uncertainties, although not inconsequential, are far less dramatic.^6^ This important conclusion, perhaps the most significant of this entire report, follows from a comparison of Adjustment No. 4 with No. 1.

## 4. Conclusion

Physics and metrology have not stood still since 1 January 1986, the cutoff date for data to be considered for inclusion in the 1986 CODATA least-squares adjustment of the constants; many new results have been reported in the intervening $4\frac{1}{2}$ years that lead to significant changes in the 1986 set of recommended values. In fact, only 5 of the 20 items of stochastic input data considered in this report (table 2) are the same as those considered in 1986: *F*, the NPL value of $\gamma \prime_{p}\left(\mathrm{hi}\right)$, the two values of µ_µ_/µ_p_, and the experimental value of *v*_Mhfs_. There have been changes in the auxiliary constant and secondary stochastic data categories as well. The most notable change in an auxiliary constant is that of the Rydberg. New measurements of *R*_∞_ imply that the 1986 recommended value is too small by 2.8 times the 1.2 × 10^−9^ relative uncertainty assigned the 1986 value. Moreover, the 1.7 × 10^−10^ relative uncertainty quoted for the most precise of these is 7 times smaller. The improvement in the secondary stochastic datum *R*, the molar gas constant, is nearly as great: The new result for *R* with its 1.7 ppm uncertainty is nearly 5 times more precise than the 1986 value with its 8.4 ppm uncertainty. Fortunately, the change implied in the 1986 value, −4.7 ppm, is only 0.6 times this uncertainty. Similar changes occur in the values and uncertainties of other constants that are dependent upon *R*, for example, the Boltzmann constant *k* and Stefan-Boltzmann constant *σ.*

Of course, the bulk of the changes in the 1986 set of recommended values arise from the stochastic input data of table 2. Our discussion of the impact of these data as given throughout this report, but especially in section 3, may be succinctly summarized as follows (see table 7): The changes in the 1986 values are generally less than twice the uncertainties of the 1986 values; shifts in the range 1.6–1.8 standard deviations are typical. The uncertainties of the 1986 values themselves are reduced by a factor of 4.7–6.9. These changes are, however, strongly dependent upon just two new stochastic input data items: the value of *W*_90_ obtained from the NPL moving coil experiment (data item 2.1, table 2); and the University of Washington-Cornell value of α^−1^ obtained from the electron magnetic moment anomaly *a*_e_ (data item 9.1). If these data items are deleted, the changes in the 1986 values are in general only 1.0–1.2 standard deviations and their uncertainties are only reduced by about a factor of 2.

The strong dependence of the changes in the 1986 recommended values and their uncertainties on just three results (counting the new value of *R*) has significant implications for the timing of the next least-squares adjustment. While the large reduction in the 1986 uncertainties arising from these three results provides some motivation for carrying out a new adjustment sooner rather than later, their dominant role must be seen as a caution sign. We believe it is of the utmost importance to obtain corroboration of each of the three results at a comparable level of uncertainty before a new set of recommended values is introduced. This is especially true of the NPL value of *W*_90_ because of its significant impact on the values of a large number of constants and because it may be argued that in view of the scatter of the data upon which it is based [52], its quoted uncertainty is somewhat optimistic.

While any work that provides added confidence in these three results would be useful and is strongly encouraged, obtaining independent values with comparable uncertainties is the obvious goal. With regard to *α*^−1^, further experimental work on measuring *a*_e_ currently underway at the University of Washington should clarify a number of possible systematic errors such as the cavity and “elevator” effects (see sec. 2.1.5). An independent value might also be available from the group working at CERN [16]. Kinoshita is continuing to check his monumental QED calculation of *a*_e_(theor), but no other group is likely to repeat this effort in the foreseeable future. Nonetheless, increased effort should be devoted to improved measurements of *Ω*_90_ and $\gamma \prime_{p}{\left(\mathrm{lo}\right)}_{90}$ in order to obtain values of α^−1^ with an uncertainty comparable with the current 0.0069 ppm uncertainty of *α*^−1^(*a*_e_), say 0.01 ppm. We therefore urge those national standards laboratories capable of carrying out calculable capacitor determinations of *Ω*_90_ and measurements of $\gamma \prime_{p}{\left(\mathrm{lo}\right)}_{90}$ to exert every possible effort to reach this level of uncertainty. (This also applies to the new Los Alamos/Yale *v*_Mhfs_ experiment and the calculation of *v*_Mhfs_(theor).) The fact that the NIST has already reported a value of *Ω*_90_ with an uncertainty of 0.024 ppm and a value of *α*^−1^ from $\gamma \prime_{p}{\left(\mathrm{lo}\right)}_{90}$ with an uncertainty of 0.037 ppm provides hope that in both cases 0.01 ppm may be achievable.

With regard to *W*_90_, as noted in section 2.2.2 Kibble and colleagues at the NPL are constructing a completely new and significantly improved moving coil apparatus to determine *W*_90_ with a relative uncertainty of ∼0.01 ppm; first results are expected in 1–2 years. The similar experiment at the NIST with the same long term uncertainty goal is also being vigorously pursued and a result for *W*_90_ with a 0.1 ppm uncertainty could be available in the same 1–2 year time period. Since a value of *W*_90_ with a 0.1 ppm uncertainty provides a value of *V*_90_, or equivalently *K*_v_, with an uncertainty of 0.05 ppm, the approximate 0.3 ppm uncertainty of the volt balance experiments carried out at the CSIRO/NML, the PTB, and the U. Zagreb (see sec. 2.2.3) would have to be reduced by about a factor of six to yield values of *K*_v_ with the same 0.05 ppm uncertainty. Similarly, the approximate 1 ppm uncertainty of the NIST and PTB/CBNM measurements of *N*_A_ would need to be reduced by about a factor of 10 to yield a value of *K*_v_ with a comparable uncertainty. While such uncertainty reductions will be difficult to achieve, we again urge those laboratories engaged in volt balance and Avogadro constant experiments to exert every possible effort to do so in view of the potential impact of the results on the next set of recommended values of the constants, as well as on replacing the kilogram [22]. This encouragement is extended also to those researchers and laboratories engaged in other approaches, for example, determining *W*_90_ by comparing mechanical and electrical energy through the levitation of a superconducting mass with a superconducting coil (see sec. 2.2.2).

With regard to the molar gas constant *R*, Mold-over and colleagues at the NIST are continuing to carry out acoustical resonator measurements that may provide added confidence in the present NIST value with its 1.7 ppm uncertainty. However, what is needed is a new version of the experiment that can take full advantage of all that has been learned in the earlier version and of related research that points the way to determining the volume of the resonator from microwave resonance measurements [78] rather than from weighing the mercury required to fill it. We hope that a new effort is initiated at the NIST in the near future and that other laboratories also consider undertaking such work.

In summary, we believe it is premature to predict when the next least-squares adjustment of the constants should be carried out. While a new set of recommended values could be introduced today with uncertainties considerably smaller than those of the 1986 set, inasmuch as the 1986 set has only been available for about 4 years, we believe for the reasons given in section 1.1 that this would be premature. That the changes in the 1986 values arising from the currently available data are not highly significant, that the data are dominated by just three new results, and that there are some annoying inconsistencies among the data, supports this view. It therefore appears best to postpone the decision as to when a new set of recommended values to replace the 1986 set should be introduced until some significant progress is made in the experimental and theoretical work just discussed. This progress will no doubt strongly influence the timing of the next adjustment. Indeed, it is conceivable that new results obtained in the next several years will suggest that the introduction of a new set of recommended values be further postponed because of unacceptable inconsistencies among the data. One point about which we are certain is that the future of this field of science cannot be predicted—the discovery of a new phenomenon with the impact of the Josephson effect or the quantum Hall effect may await us just next year!

## Figures and Tables

**Table 1:** Summary of values of the Rydberg constant *R*_∞_

Authors, publication date, reference	Transition	Reported value and uncertainty (*R*_∞_/m^−1^−10 973 731)	Relative uncertainty (parts in 10^10^)
Cohen and Taylor CODATA 1986 recommended value [2]		0.534(13)	12
Zhao et al., 1986 [23]	H, D, 2*S*−3*P*	0.5689(71)	6.5
Biraben et al., 1986 [24]	H, D, 2*S*−8,10*D*	0.5692(60)	5.5
Beausoleil et al., 1987 [28]	H, 1*S*−2*S*	0.5715(67)	6.1
Zhao et al., 1989 [25]	H, D, 2*S*−4*P*	0.5731(29)	2.6
Boshier et al., 1989 [26]	H, D, 1*S*−2*S*	0.5731(31)	2.8
McIntyre et al., 1989 [27]	H, 1*S*−2*S*	0.5686(78)	7.1
Biraben et al., 1989 [29,30]	H, D, 2*S*−8,10,12 *D*	0.5709(18)	1.7

**Table 2:** Summary of principal items of stochastic input data

Data type and item no.	Measurement date	Identification	Value	Relative uncertainty (ppm)
1. Ohm, *Ω*_90_			Ω	
1.1	1985–1988	CSIRO/NML (Australia)	1.000 000 092(66)	0.066
1.2	1985–1988	NPL (UK)	1.000 000 085(54)	0.054
1.3	1983–1988	NIST (USA)	1.000 000 009(24)	0.024
2. Watt, *W*_90_			W	
2.1	1987–1988	NPL (UK)	0.999 999 903(136)	0.14
2.2	1988	NIST (USA)	1.000 000 24(133)	1.33
3. Volt, *V*_90_			V	
3.1	1983	CSIRO/NML (Australia)	0.999 999 975(269)	0.27
3.2	1989	PTB (FRG)	1.000 000 027(274)	0.27
4. Faraday, *F*			*C*_90_/mol	
4.1	1975–1984	NIST (USA)	96 485.384(128)	1.33
5. Proton gyromagnetic ratio, $\gamma \prime_{p}$, low			10^4^ s-1/*T*_90_	
5.1	1986–1988	NIST (USA)	26 751.5427(29)	0.11
5.2	1987	VNIM (USSR)	26 751.5630(96)	0.36
6. Proton gyromagnetic ratio, $\gamma \prime_{p}$, high			10^4^ C_90_/kg	
6.1	1973–1974	NPL (UK)	26 751.503(27)	1.01
7. Proton gyromagnetic ratio, $\gamma \prime_{p}$			10^4^ s^−l^/T	
7.1	1988	NIM (PRC)	26 751.541(23)	0.86
7.2	1985	ASMW (GDR)	26 751.427(21)	0.80
8. Avogadro constant, *N*_A_			10^23^ mol^−1^	
8.1	1974–1989	NIST (USA)	6.022 1315(72)	1.19
8.2	1982–1989	PTB/CBNM (FRG, Belg.)	6.022 1341(66)	1.10
9. Inverse fine-structure constant, *α*^−1^				
9.1	1987–1990	U. Wash./Cornell (USA)	137.035 992 22(94)	0.0069
9.2	1989	PTB (FRG)	137.035 993(27)	0.20
10. Muon magnetic moment, µ*_µ_*/µ_p_				
10.1	1982	Los Alamos/Yale (USA)	3.183 3461(11)	0.36
10.2	1982	SIN Switzerland	3.183 3441(17)	0.53
11. Muonium hyperfine interval, *v*_Mhfs_			kHz	
11.1	1982	Los Alamos/Yale (USA)	4 463 302.88(62)	0.14

**Table 3:** Summary of values of *K*_Ω_ = (*R*_K_/*R*_K−90_) = µ_0_*c*α^−1^/2*R*_K−90_ taken directly from or derived from the stochastic data of table 2 (the 1986 value excepted)

Identification	Value and uncertainty Δ = (*K*_Ω_−1) × 10^6^
1. α^−1^(*a*_e_) (data item 9.1)	−0.0328±0.0069
2. *Ω*_90_, NIST (data item 1.3)	0.009±0.024
3. *α*^−1^ from NIST $\gamma \prime_{p}\left(\mathrm{lo}\right)$ (from data item 5.1)	−0.093±0.037
4. *Ω*_90_, NPL (data item 1.2)	0.085±0.054
5. *Ω*_90_, CSIRO/NML (data item 1.1)	0.092±0.066
6. α^−1^ from VNIIM $\gamma \prime_{p}\left(\mathrm{lo}\right)$ (from data item 5.2)	−0.35±0.12
7. α^−1^(Mhfs) (from data items 10.1, 10.2, 11.1)	−0.03±0.16
8. *α*^−1^(*h/m*_n_) (data item 9.2)	−0.03±0.20
CODATA 1986 recommended value	−0.052±0.045

**Table 4:** Summary of values of *K*_v_*=K*_J−90_*/K*_J_*=K*_J−90_*/*(2*e/h*) taken directly from or derived from the stochastic input data of table 2 (the 1986 value excepted)

Identification	Value and uncertainty Δ = (1−*K*_v_) × 10^6^
1. *K*_v_ from NPL *W*_90_ and *Ω*_90_ via α^−1^(*a*_e_) (from data items 2.1 and 9.1)	0.065±0.068
2. *V*_90_, CSIRO/NML (data item 3.1)	0.025±0.269
3. *V*_90_, PTB (data item 3.2)	−0.027±0.274
4. 2*e/h* from NPL $\gamma \prime_{p}\left(\mathrm{hi}\right)$ and *α*^−1^(*a*_e_) (from data items 6.1 and 9.1)	−0.62±0.50
5. 2*e/h* from PTB/CBNM *N*_A_ and *α*^−1^(*a*_e_) (from data items 8.2 and 9.1)	−0.68±0.55
6. 2*e/h* from NIST *N*_A_ and *α*^−1^(*a*_e_) (from data items 8.1 and 9.1)	−0.89±0.60
7. *K*_v_ from NIST *W*_90_ and *Ω*_90_ via *α*^−1^(*a*_e_) (from data items 2.2 and 9.1)	−0.10±0.67
8. 2*e/h* from NIST *F* and *α*^−l^(*a_e_*) (from data items 4.1 and 9.1)	0.19±0.67
9. 2*e/h* from ASMW $\gamma \prime_{p}$ and *α*^−1^(*a*_e_) (from data items 7.2 and 9.1)	−4.11+0.80
10. 2*e/h* from NIM $\gamma \prime_{p}$ and *α*^−1^(*a*_e_) (from data items 7.1 and 9.1)	0.15±0.86
CODATA 1986 recommended value	−0.47±0.30

**Table 5:** The 11 distinct observational equations for the least-squares analysis of the stochastic data of table 2 taking *K*_Ω_, *K*_v_, and µ_µ_/µ_P_ as the unknowns

1. *Ω*_90_=*K*_Ω_ *Ω*
2. $W_{90}=K_{\Omega }^{-1}K_{V}^{2}\;W$
3. *V*_90_ = *K*_V_V
4. $F_{90}=\frac{\mu_{0}^{2}c^{3}K_{J-90}M_{p}}{16R_{K-90}^{2}R_{\infty }\left(m_{p}/m_{e}\right)}K_{\Omega }^{-1}\;K_{V}^{-2}$
5. $\gamma \prime_{p}{\left(\mathrm{lo}\right)}_{90}=\frac{\mu_{0}^{2}c^{3}K_{J-90}\left(\mu \prime_{p}/\mu_{B}\right)}{16R_{K-90}^{2}R_{\infty }}K_{\Omega }^{-3}$
6. $\gamma \prime_{p}{\left(\mathrm{hi}\right)}_{90}=\frac{\mu_{0}^{2}c^{3}K_{J-90}\left(\mu \prime_{p}/\mu_{B}\right)}{16R_{K-90}^{2}R_{\infty }}K_{\Omega }^{-1}K_{V}^{-2}$
7. $\gamma \prime_{p}=\frac{\mu_{0}^{2}c^{3}K_{J-90}\left(\mu \prime_{p}/\mu_{B}\right)}{16R_{K-90}^{2}R_{\infty }}K_{\Omega }^{-2}K_{V}^{-1}$
8. $N_{A}=\frac{\mu_{0}^{2}c^{3}K_{J-90}^{2}M_{p}}{32R_{K-90}R_{\infty }\left(m_{p}/m_{e}\right)}K_{\Omega }^{-1}K_{V}^{-2}$
9. $\alpha^{-1}=\frac{2R_{K-90}}{\mu_{0}\;c}K_{\Omega }$
10. µ_µ_/µ_p_ = µ_µ_/µ_p_
11. $\begin{array}{c} \nu_{\mathrm{Mhfs}}=\frac{4\mu_{0}^{2}c^{3}R_{\infty }\left(\mu_{p}/\mu_{B}\right)q}{3{R^{2}}_{K-90}{\left(1+m_{e}/m_{\mu }\right)}^{3}}K_{\Omega }^{-2}\left(\mu_{\mu }/\mu_{p}\right)\\ q=1.000\;957\;65\left(14\right) \end{array}$

**Table 6:** Summary of results of four least-squares adjustments involving the stochastic input data of table 2

	Adjustment No. 1	Adjustment No. 2 (NPL *W*_90_ deleted)	Adjustment No. 3 (α^−1^(*a*_e_) deleted)	Adjustment No. 4 (NPL *W*_90_, α^−l^(*a*_e_) deleted)
*K*_Ω_−1 (ppm)	−0.0285±0.0064	−0.0287±0.0064	0.000±0.018	−0.001±0.018
1−*K*_v_ (ppm)	0.026±0.062	−0.16±0.15	0.015±0.062	−0.15±0.15
µ_µ_/µ_p_	3.183 345 48(40) (0.13 ppm)	3.183 345 48(40) (0.13 ppm)	3.183 345 63(41) (0.13 ppm)	3.183 345 63(41) (0.13 ppm)
Total data items deleted	5.2, 7.2	NPL *W*_90_ (2.1), 5.2, 7.2	α^−1^(*a*_e_) (9.1), 5.2, 7.2	NPL *W*_90_ (2.1), α^−1^(*a*_e_) (9.1), 5.2, 7.2
*χ*^2^; *v*; *R*_B_	20.7; 15; 1.17	18.9; 14; 1.16	17.7; 14; 1.12	16.2; 13; 1.12
$P_{\chi^{2}}\left(\chi^{2}|\nu \right)$	0.15	0.17	0.22	0.24

**Table 7:** Changes in the 1986 recommended values and uncertainties of a representative group of constants implied by the new results reported since the completion of the 1986 adjustment

Quantity	1 std. dev. uncer. of 1986 recom. value (*σ*_86_) in ppm	ppm change in 1986 recommended value (δ), corresponding number of standard deviations (δ/*σ*_36_), new ppm uncertainty (*σ*_90_), and ratio of 1986 uncertainty to new uncertainty^a^ (*σ*_86_/*σ*_90_)															
		Adjustment No. 1				Adjustment No. 2 (NPL *W*_90_ deleted)				Adjustment No. 3 (α^−1^(*a*_e_) deleted)				Adjustment No. 4 (NPL *W*_90_, *α*^−1^(*a*_e_) deleted)			
	
	*σ*_86_	*δ*	*δ/σ*_86_	*σ*_90_	*σ*_86_/*σ*_90_	*δ*	δ/*σ*_86_	*σ*_90_	*σ*_86_/*σ*_90_	*δ*	*δ/σ*_86_	*σ*_90_	*σ*_86_/*σ*_90_	*δ*	δ/*σ*_86_	*σ*_90_	*σ*_86_*/σ*_90_
*H*	0.60	−1.02	−1.7	0.12	4.8	−0.65	−1.1	0.30	2.0	−1.03	−1.7	0.12	4.8	−0.69	−1.2	0.31	2.0
*e*	0.30	−0.52	−1.7	0.062	4.9	−0.34	−1.1	0.15	2.0	−0.54	−1.8	0.063	4.8	−0.37	−1.2	0.15	2.0
*m*_e_	0.59	−0.97	−1.6	0.12	4.7	−0.60	−1.0	0.30	1.9	−0.92	−1.6	0.13	4.6	−0.59	−1.0	0.30	1.9
*α*^−1^,*R*_K_	0.045	0.024	0.5	0.0064	6.9	0.024	0.5	0.0064	6.9	0.053	1.2	0.018	2.5	0.051	1.1	0.018	2.5
*N*_A_	0.59	0.97	1.6	0.12	4.7	0.60	1.0	0.30	1.9	0.92	1.6	0.13	4.6	0.58	1.0	0.30	1.9
*F*	0.30	0.45	1.5	0.064	4.7	0.26	0.9	0.15	2.0	0.38	1.2	0.075	4.0	0.21	0.7	0.16	1.9
*R*	8.4	−4.7	−0.6	1.7	4.9	−4.7	−0.6	1.7	4.9	−4.7	−0.6	1.7	4.9	−4.7	−0.6	1.7	4.9
*k*	8.5	−5.7	−0.7	1.7	4.9	−5.3	−0.6	1.7	4.9	−5.6	−0.7	1.7	4.9	−5.3	−0.6	1.7	4.9
*σ*	34	−20	−0.6	6.9	4.9	−19	−0.6	6.9	4.9	−19	−0.6	6.9	4.9	−19	−0.6	6.9	4.9
*K*_J_	0.30	0.50	1.7	0.062	4.8	0.32	1.1	0.15	1.9	0.49	1.6	0.062	4.7	0.32	1.1	0.15	1.9
µ_B_	0.34	−0.57	−1.7	0.064	5.2	−0.39	−1.2	0.15	2.2	−0.65	−1.9	0.078	4.3	−0.48	−1.4	0.16	2.1
λ_c_	0.089	−0.051	−0.6	0.013	6.9	−0.051	−0.6	0.013	6.9	−0.109	−1.2	0.036	2.5	−0.106	−1.2	0.036	2.5
$\gamma \prime_{p}$	0.30	0.45	1.5	0.064	4.7	0.26	0.9	0.15	2.0	0.38	1.2	0.075	4.0	0.21	0.7	0.16	1.9
*m*_µ_*/m*_e_	0.15	0.00	0.0	0.13	1.2	0.00	0.0	0.13	1.2	−0.05	−0.4	0.13	1.1	−0.05	−0.3	0.13	1.1

a See section 3.2 for a full description of Adjustment Nos. 1–4.

**Table 8:** Changes in the 1986 recommended values and uncertainties of a representative group of constants implied by the three dominant new results alone: the value of *h* obtained from the NPL determination of *W*_90_, the University of Washington-Cornell value of *α*^−1^(*a*_e_), and the NIST value of *R*

Quantity	1 std. dev. uncer. of 1986 recom.value (*σ*_86_) in ppm	ppm change in 1986 recommended value (δ), corresponding number of standard deviations (δ/*σ*_86_), new ppm uncertainty (*σ*_90_), and ratio of 1986 uncertainty to new uncertainty (*σ*_86_/*σ*_90_)			

	σ_86_	δ	δ/σ_86_	*σ*_90_	*σ*_86_/*σ*_90_
*h*	0.60	−1.10	−1.8	0.14	4.4
*e*	0.30	−0.56	−1.8	0.068	4.5
*m*_e_	0.59	−1.05	−1.8	0.14	4.3
*α*^−1^,*R*_K_	0.045	0.020	0.4	0.0069	6.5
*N*_A_	0.59	1.05	1.8	0.14	4.3
*F*	0.30	0.49	1.6	0.070	4.3
*R*	8.4	−4.7	−0.6	1.7	4.9
*k*	8.5	−5.8	−0.7	1.7	4.9
*σ*	34	−20	−0.6	6.9	4.9
*K*_J_	0.30	0.54	1.8	0.068	4.4
µ_B_	0.34	−0.60	−1.8	0.070	4.8
λ_c_	0.089	−0.043	−0.5	0.014	6.5
$\gamma \prime_{p}$	0.30	0.50	1.6	0.070	4.3
*m_µ_/m*_e_	0.15	0.00	0.0	0.13	1.2

## Footnotes

1 CODATA, the Committee on Data for Science and Technology, was established in 1966 as an interdisciplinary committee of the International Council of Scientific Unions. It seeks to improve the compilation, critical evaluation, storage, and retrieval of data of importance to science and technology.

2 The current members of the CODATA Task Group on Fundamental Constants are T. J. Quinn (Chairman), E. R. Cohen, T. Endo, B. Kramer, B. A. Mamyrin, B. N. Oleinik, B. W. Petley, H. Preston-Thomas, and B. N. Taylor.

3 Throughout, all uncertainties are one-standard-deviation estimates.

4 To simplify comparisons, we quote *R*_∞_ − 10 973 731 m^−1^ rather than *R*_∞_ itself.

5 The choice of these three variables for the least squares analysis is not unique; we could have taken, for example, *α, h*, and *µ_µ_/µ*_p_ equally as well.

6 See the Appendix, section 5.2, for a brief discussion of the changes in the 1986 set of recommended values that arise from these three results alone.

## 5. Appendix

### 5.1 Laboratory Abbreviations

The following are the laboratory abbreviations used throughout this paper.

#### Abbreviations

ASMW: Amt für Standardisierung, Messwesen und Warenprüfung, Berlin, GDR
BIPM: Bureau International des Poids et Mesures, Sèvres, France
CBNM: Central Bureau for Nuclear Measurements, Geel, Belgium
CSIRO/NML: Commonwealth Scientific and Industrial Research Organization, Division of Applied Physics, National Measurement Laboratory, Lind-field, Australia
ETL: Electrotechnical Laboratory, Tsukuba, Japan
KhGIMIP: Kharkov State Scientific Research Institute of Metrology, Kharkov, USSR
LCIE: Laboratoire Central des Industries Électriques, Fontenay-aux-Roses, France
NIM: National Institute of Metrology, Beijing, PRC
NIST: National Institute of Standards and Technology (formerly National Bureau of Standards or NBS), Gaithersburg, MD, USA
NPL: National Physical Laboratory, Teddington, UK
NRLM: National Research Laboratory of Metrology, Tsukuba, Japan
PTB: Physikalisch-Technische Bundesanstalt, Braunschweig, FRG
SIN: Swiss Institute for Nuclear Research, Villigen, Switzerland
U. Zagreb: Faculty of Electrical Engineering, University of Zagreb, Yugoslavia
VNIIM: All-Union Scientific Research Institute of Metrology (Mendeleev Institute of Metrology), Leningrad, USSR

### 5.2 Effect of the Three Dominant New Results Alone

It is of interest to calculate the changes in the 1986 recommended values and uncertainties of the same representative group of constants listed in table 7 arising from the three dominant new results alone: *h* =6.626 068 21(90) × 10^−34^ J s (0.14 ppm) as obtained from the NPL Measurement of *W*_90_ (data item 2.1 of table 2) using eqs (12) and (13); *α*^−1^(*a*_e_) (eq (21a), data item 9.1); and the NIST value of *R*, eq (27). Using relations such as *e* = (2*h*/*µ*_0_*cα*^−1^)^1/2^, *K*_J_ = 2*e*/*h* = (8/*µ*_0_*ch*α^−1^)^1/2^, and eqs (25) and (26) for *k* and *σ*, yields the results given in table 8. A comparison of table 8 with Adjustment No. 1 of table 7 again shows that these three results alone account for most of the observed changes in the 1986 recommended values and their uncertainties. (The value µ_µ_/µ_p_ = 3.183 345 46(40) (0.13 ppm) used to calculate the ratio *m_µ_/m*_e_ is the weighted mean of the indirect “muonium” value µµ/µ_p_ = 3.183 345 46(45) (0.14 ppm) as obtained from *α*^−1^(*a*_e_), the experimental value of *v*_Mhfs_ (data item 11.1), and the theoretical expression for *v*_Mhfs_ (eq (11) of table 5, see the discussion in sees. 3.2 and 3.3.); and the weighted mean of the two direct measurements µ_µ_/µ_p_ = 3.183 345 47(95) (0.30 ppm) (data items 9.1 and 9.2).)
